# An Internet-Based, Peer-Delivered Messaging Intervention for HIV Testing and Condom Use Among Men Who Have Sex With Men in India (CHALO!): Pilot Randomized Comparative Trial

**DOI:** 10.2196/16494

**Published:** 2020-04-16

**Authors:** Viraj V Patel, Shruta Rawat, Alpana Dange, Corina Lelutiu-Weinberger, Sarit A Golub

**Affiliations:** 1 Division of General Internal Medicine, Department of Medicine Albert Einstein College of Medicine Montefiore Health System Bronx, NY United States; 2 The Humsafar Trust Mumbai India; 3 François-Xavier Bagnoud Center School of Nursing Rutgers Biomedical and Health Sciences Newark, NJ United States; 4 Department of Psychology Hunter College City University of New York New York, NY United States

**Keywords:** HIV, internet, mHealth, eHealth, men who have sex with men, sexual minority, LGBT, India, intervention, prevention, mobile phone

## Abstract

**Background:**

Leveraging internet-based communication tools (eg, messaging apps, SMS text messaging, and email) may be an effective avenue for delivery of HIV prevention messages to men who have sex with men (MSM) in India, but there are limited models for such internet-based interventions.

**Objective:**

The CHALO! pilot was an online educational and behavioral intervention aimed to determine the feasibility, acceptability, and preliminary impact of a peer-delivered, internet-based messaging intervention for HIV testing and consistent condom use for MSM in India. The messages addressed barriers to HIV testing and condom use and were theoretically based on the information-motivation-behavioral skills model.

**Methods:**

Between February and March 2015, we recruited, enrolled, and randomized 244 participants via online advertisements on mobile dating apps and Facebook. Eligible men (18 years or older, sexually active with other men, and self-reported HIV-negative or unknown status) were randomized to receive educational and motivational messages framed as either approach (ie, a desirable outcome to be achieved) or avoidance (an undesirable outcome to be avoided) over 12 weeks via internet-based messaging platforms. Participants completed online surveys at baseline and immediately postintervention.

**Results:**

Participants were similar across arms with respect to sociodemographic and behavioral characteristics. Over 82.0% (200/244) of participants were retained (ie, viewed final messages), and 52.3% (130/244) of them completed the follow-up survey. Of those completing the follow-up survey, 82.3% (107/130) liked or strongly liked participating in CHALO!. The results showed a significant increase in self-reported HIV testing in the past 6 months from baseline to follow-up (41/130, 31.5% to 57/130, 43.8%; *P*=.04). When including those who reported intentions to test, this percentage increased from 44.6% (58/130) at baseline to 65.4% (85/130) at follow-up (*P*<.01). When examining intentions to test among those without prior HIV testing, intentions increased from 32% (16/50) of the sample at baseline to 56% (28/50) of the sample at follow-up (*P*=.02). Condom use during anal sex did not significantly change from baseline to follow-up. HIV testing and condom use did not significantly differ between approach and avoidance conditions at follow-up.

**Conclusions:**

As one of the first studies of an online HIV prevention intervention for Indian MSM, CHALO! was feasible to implement by a community-based organization, was acceptable to participants, and demonstrated potential to improve HIV testing rates.

## Introduction

### Background

India has the third largest population of people living with HIV globally, with an estimated 2.1 million infected persons, and more than 80,000 new infections occurring annually [1]. Globally and in India, HIV disproportionately affects men who have sex with men (MSM), who are designated a key priority population by the Indian health ministry for targeted HIV prevention interventions [2]. HIV prevalence among MSM in India is 10 to 15 times higher than in the general population (4.3% vs 0.3%, respectively) [1], and although MSM are conservatively estimated to make up less than 2% of the population, they comprise over 20% of HIV-infected individuals [1,2]. Thus, current interventions for Indian MSM have been limited in their reach and impact [3], partly because of the social stigma associated with same sex behaviors and marginalization [4,5]. The cultural emphasis on heterosexual marriage and traditional family structure, coupled with the constant fear of one’s sexuality being *outed* [6-9], drive many MSM *underground* and out of the purview of various HIV interventions currently being implemented. To reduce the burden of HIV among Indian MSM, rapid development and wide-scale dissemination of interventions promoting effective prevention strategies are urgently needed.

Currently, over 430 million individuals in India access the internet, with 95% using mobile devices. Although access is currently greater among individuals younger than 35 years and those with higher incomes [10], these gaps are quickly closing, given the rapid decline in the cost of smartphones and data plans and the increasing availability of free Wi-Fi spots. Globally and in India, MSM are increasingly using internet-based communication technologies (ICTs; eg, Facebook, geolocation-based mobile dating apps, and email)—to socialize, seek sexual and romantic partners, and find a sense of community [11-14]. Conducting traditional face-to-face HIV prevention outreach for MSM can be challenging within stigmatized settings, but given the increasing use of technology by MSM to find partners and supportive social networks, ICTs now allow for an unprecedented opportunity to engage Indian MSM into HIV prevention, linkage to care, and other support services. Data indicate that the populations that can be reached online are in dire need of increased access to prevention services. Recent studies of MSM reached online in India have found that, among sexually active MSM, over 50% had never had an HIV test, a quarter had not been tested in more than 12 months, and between 40% and 80% were not out to others about their sexuality [15,16]. Thus, ICT-based interventions for MSM could dramatically improve the health of MSM in India and globally by helping support behavior change for HIV prevention (eg, HIV testing and condom use) [13].

International organizations, including India’s National AIDS Control Organization, recognize the public health potential of ICTs and have called for the development and implementation of ICT-based HIV prevention strategies [2]. Besides evidence of their being acceptable to MSM, ICTs also offer considerable scalability and efficiency of wide reach with high impact potential, even with relatively low-intensity interventions [17,18]. Technology-based, peer-led approaches could be used to enhance efforts by community-based and other organizations for dissemination of health messages and service availability [19]. Thus, rather than an alternative medium for implementation of existing interventions designed for face-to-face contact, social media may be a *game changer* to engage MSM in India [20]. A meta-analysis found that social media interventions were effective in increasing HIV testing; however, none of the studies were conducted in a low-income country [21]. Two recent systematic reviews describing internet-based interventions for HIV care continuum found diverse models targeting HIV testing and prevention; however, most (over 85%) were in well-resource settings [22,23]. Few effective, scalable, and low-cost ICT-based interventions targeting HIV testing and prevention exist for low-income countries [24-27], and no published data are available on the effectiveness of ICT-based approaches in India or other South Asian countries.

In addition to the paucity of data about internet-based interventions in low-income settings for any population, there is little empirical data to guide health communication, that is, messaging, for online dissemination to increase HIV testing and condom use. Two messaging approaches, often called *frames* are widely used in health communication: the first, called *approach* or *gain framed*, highlights the benefits of engaging in a specific health behavior and the second, called *avoidance* or *loss framed*, focuses on negative consequences. Metanalytic reviews have indicated that gain-framed messaging is more effective in promoting prevention behavior, but loss-framed messaging may be more effective in promoting screening or illness detection behavior [28-30]. However, it is unclear which framing strategy is most effective when promoting a comprehensive approach to HIV prevention that includes promoting both HIV testing and condom use. Prior research from a high-income country (United States) has found mixed results with regard to condom use intentions [31,32] and HIV testing behaviors [33]. However, to our knowledge, no published data exist with regard to the framing effects of health messages for HIV testing and condom use for MSM or for any other populations in India or other low-income countries.

### Objectives

To help close the gap in the use of ICT for public health purposes in India and address the high HIV prevention needs of Indian MSM, the CHALO! (*Let’s Go!*) pilot study developed and tested the feasibility, acceptability, and preliminary impact of an ICT-based HIV prevention intervention to increase HIV testing and consistent condom use among MSM reached on internet-based social and dating platforms in Mumbai, India. CHALO! also tested whether prevention messages using an approach frame (ie, messages highlighting a desirable outcome to be achieved or benefits of engaging in testing and condom use) were more effective in increasing HIV testing and consistent condom use behaviors compared with messages using an avoidance frame (ie, HIV infection as an outcome to be avoided or consequences of not engaging in a behavior). Our central hypothesis was that a peer-delivered, ICT-based behavioral intervention can efficiently identify and reach sexually active Indian MSM, enroll them into an exclusively online study, motivate them to seek in-person health services (ie, HIV testing), and modify health promotion behaviors (increase consistent condom use). This study was a peer-delivered intervention that recruited participants online and then disseminated HIV prevention messages via internet-based messaging platforms.

## Methods

### Study Design and Overview

The study was conducted in partnership with the Humsafar Trust (HST), one of India’s largest community-based organizations based in Mumbai, providing culturally sensitive clinical and social services to sexual and gender minority populations. CHALO! was a two-arm, parallel, randomized (1:1 randomization) comparative effectiveness trial comparing two message-framing strategies (avoidance- and approach-framed messages) to promote HIV testing and consistent condom use. Messages were delivered by four peer-outreach staff (two per arm) via email, a private Facebook group, or WhatsApp (as chosen by the participant). Participants received the intervention messages twice a week for 12 weeks. In addition to the messaging, other intervention components were (1) the ability to communicate with the peer outreach staff via their chosen messaging modality and (2) a mobile-friendly Web page containing information on accessing MSM-sensitive, free HIV testing in Mumbai; free condoms and instructions on use; and a listing of available services for MSM at HST (eg, counseling, sexually transmitted infection [STI] treatment, and support groups). We used self-administered online surveys at baseline and 12-week postintervention for study assessments. The study was approved by the Humsafar Trust’s and Albert Einstein College of Medicine’s institutional review board.

### Setting

The study took place online, between February and June 2015, targeting MSM living in Mumbai—India’s largest city with a population of over 18 million, and a city with one of the highest HIV burdens in India. Mumbai accounted for over 19,000 new HIV diagnoses in 2016-2017 [1,2], with the prevalence of HIV among MSM estimated to be 7%. At the time of the study, Mumbai had high internet connectivity, with an abundance of free or low-cost Wi-Fi spots, low-cost internet cafes, and mobile service providers offering internet and data plans for mobile phones at a relatively low cost. For this pilot, we recruited participants from two of India’s most used MSM-specific dating sites (which have now become the most commonly used avenues for MSM meets in urban India [34,35]) and from HST-operated Facebook pages. HST has three drop-in centers and a central office in the three major subdivisions of Mumbai, where HIV and STI testing, sexual health and psychosocial counseling, and linkage-to-care services are available. At the time of the study, HST was the only established community organization providing such services to MSM.

### Participants

Eligible individuals were aged at least 18 years, identified as male, reported anal sex with another male partner in the past 2 years, lived in Mumbai, were fluent in either English or Hindi, self-reported being HIV negative or unaware of their status (ie, never tested or never received results), and provided a valid contact (email, mobile phone number, or Facebook ID—validated by a response to a confirmation message). Individuals were excluded if they reported being a staff member or any type of outreach worker for HST. Participants were screened into the study using an online screening survey.

### Theoretical Basis

The CHALO! pilot drew on theories from health psychology (information-motivational-behavioral [IMB] skills theory [36,37]) and health communication (Prospect Theory) [38]. The IMB model posits that fostering information acquisition, increasing motivation, and enhancing behavioral skills are needed to change behaviors (eg, HIV testing and condom use). We used the IMB model to inform the specific message contents used in the intervention. We next used the Prospect Theory to frame the messages for each arm. Framing effects are a central tenet of the Prospect Theory [39], which posits that decision making is affected by the manner in which choices are presented; for example, behavioral science has demonstrated differences in health screening behaviors and other health-related decisions, when options for engaging in a health-related activity are framed in terms of potential benefits (*gain* frame) compared with potential harm (*loss* frame [39-41]). As past research suggests a significant impact of messaging framing on HIV testing behaviors [33], we incorporated tenets of this theory to help ensure that CHALO! messages were framed in a manner that would promote optimal decision making and behavior change with regard to HIV testing and consistent condom use. Messages in CHALO! were framed to either an *approach* frame (ie, highlighting a desirable outcome to be achieved or benefits of engaging in HIV testing or consistent condom use) or to an *avoidance* frame (ie, focusing attention on a negative outcome to be avoided or consequences of not engaging in HIV testing or not using condoms) [29,33].

### Intervention Development

We used a participatory process with an interdisciplinary team at HST to develop all components of the intervention in an iterative process over a 3-month period. The core team members at HST consisted of 8 individuals: 2 community-based researchers, 2 HIV testing and counseling staff members, an HIV-positive peer patient navigator, and 3 peer outreach workers experienced in using MSM dating websites and apps for outreach to MSM in Mumbai. This intervention development team informed all aspects of the study including study design, participant eligibility, recruitment and retention, study measures, intervention implementation, and evaluation.

### Target Selection and Message Development

The intervention target selection (ie, which barriers to address) and message development and refinement process was a community-led multiphase and iterative process occurring over a 2-day workshop with an interdisciplinary team: 3 facilitators (HST research staff members experienced in conducting HIV-related trainings) and 10 participants (HST’s community advisory board members and peer outreach workers experienced in outreach and care linkage for >5 years and with online MSM dating apps), and HIV counseling and testing staff. The members had diverse sexual identities common in India (gay, bisexual, *kothi*, and *panthi)* [42,43], genders (male, female, and *hijra or transgender individuals),* and demographic characteristics (with regard to education, age, and primary language used (English or Hindi). Three members of the group were people living with HIV.

We used *open space technology* to facilitate communication and participation by all workshop members [44]. Open space technique is a process that has been used across disciplines to help ensure inclusion of diverse attitudes and experiences and has been used to facilitate identification of challenges or barriers to a task or behavior (eg, HIV testing) and identification of potential solutions to overcome them. For this study, workshop members first identified challenges to HIV testing and consistent condom use, and then mapped these to the IMB domains (ie, the targets). The following targets were identified within the IMB model:

- *Information*: information about HIV transmission and prevention with condoms and logistical information (eg, testing locations and hours)
- *Motivation*: risk perception and stigma
- *Behavioral skills*: how to access or make an appointment for free testing

Next, after receiving a brief orientation to *approach* and *avoidance* messaging frames, participants in small groups developed short social marketing messages that could be disseminated online addressing the above-identified targets or provided solutions for overcoming the barrier (eg, a webpage vetted to be MSM friendly listing free HIV testing centers, which addressed lack of knowledge about safe HIV testing venues). Workshop participants developed between three and five messages for every identified target for both approach and avoidance frames. Thus, we developed approximately 25 to 30 messages for each message frame (approach and avoidance). All messages were transcreated into English or Hindi based on the original language (eg, messages initially developed in Hindi were then transcreated into English, and conversely, from English into Hindi). We used transcreation (as opposed to translation) to retain the essence of the original message [45], while the text was then refined using a consensus approach to further ensure comprehension and equivalency in meaning, sentiment, and framing. Next, 30 peer staff and MSM community members at the HST drop-in center (not involved in the workshop) voted for their favorite top 3 messages for each factor in each of the approach and avoidance frames. Finally, we selected the top 1 or 2 messages receiving the most votes for each target within each frame for use in the intervention, resulting in 15 messages for each arm: 8 messages focused on HIV testing and 7 messages focused on condom use. Here are two examples of messages used (avoidance and approach):

It doesn’t matter if you sleep with only 4 or 5. It only takes one. Not using condoms puts you at risk for HIV. Avoid HIV by using condoms!

Whether you ride from the front seat or back seat, you both need a helmet. Use a condom either way. Keep yourself and your partner healthy!

### Peer Recruitment and Training

HST research staff selected 4 MSM peer outreach workers for the intervention who were fluent in Hindi and English and reported comfort and experience with using online dating apps, Facebook, and email and not involved with development of the messages. Chosen peers had previously received training in HIV-related communication and community engagement and were experienced in HIV-related outreach in Mumbai. For this pilot, the peer outreach worker received additional specific training on online research ethics, maintaining confidentiality and privacy, and communicating via online tools. Two peer outreach workers were randomly assigned to each arm. Peers were then randomly assigned to serve as the online peer outreach worker for half the participants within their assigned arm. Each peer was responsible for sending intervention messages to their assigned participants and to communicate with participants if and when a participant chose to initiate any communication. There was no cross-arm communication from the peer outreach workers to participants in the arm to which they were not assigned.

### Intervention Procedures

First, from February to March 2015, recruitment advertisements were disseminated on a popular MSM-specific dating website, a geosocial networking mobile app, and on HST-operated Facebook pages. Potential participants clicked through the ads to complete an online consent and eligibility screener; if eligible, they automatically continued to the baseline survey. After confirming the contact information provided, participants were randomized 1:1 to either the approach- or avoidance-framed conditions.

Next, from March to June, 2015, the 4 peer outreach workers (two per arm) sent a standardized introductory message via the participant’s chosen communication modality (ie, email, WhatsApp, or private Facebook group), followed by intervention messages 2 or 3 times per week for 12 weeks. Each set of 15 messages was sent out twice over the 12 weeks to help ensure that participants viewed them and reinforce the information contained within the messages. Thus, after the first set of 15 messages were sent out, another round of the same 15 messages was sent again. Messages for all participants were sent by the peer outreach worker on the same days and times each week. Participants were also able to communicate with their assigned peer outreach worker for any reason via multiple modalities (text message, email, Facebook Messenger, or phone call), but only if the participant initiated the contact. This was to avoid being overly intrusive and prevent potential intervention fatigue based on input from the peer staff. Participants then received a final intervention message and a personal link to the follow-up survey. All messages were sent with arm-specific links to the study webpage, with additional information about HIV testing, condom use, and HST services. We compensated participants with Amazon India vouchers worth INR 300 (approximately US $4.75) on successful completion of baseline survey and INR 400 (approximately US $6.30) on successful completion of the final follow-up assessment.

### Measures

#### Sociodemographic Characteristics

At baseline, we collected information on age, monthly income, preferred language (indicated by language of survey taken—English or Hindi), and an account of household members.

#### Sexual Identity and Behaviors

We assessed sexual identity with mutually exclusive categories often used in India (*panthi*, *kothi*, double decker, gay or homosexual, bisexual, and straight or heterosexual [9-11]), but because very few respondents selected *panthi*, *kothi*, or double decker, we collapsed these categories into gay or homosexual. We asked about participants’ level of outness, whether they had sex with or were attracted to men (none, some, or most), if they had a primary male sexual partner (Yes or No), and the number of male sexual partners in the past year.

#### HIV Testing

To assess the HIV testing outcomes, we asked at baseline and follow-up, “When was your last HIV test?” with response categories of *less than 1 month ago*, *2 to 6 months ago*, *7 to 12 months ago*, *more than 12 months ago*, and *never*. We then dichotomized the responses for analysis to 6 months vs all others based on the recommended testing guidelines for MSM [46]. To ascertain testing intentions during and immediately after the intervention ended, we asked, “Do you intend to test in the next 3 months?” (at baseline) and “Do you intend to test in the next month?” (at follow-up) with the answer options of yes or no.

#### Consistent Condom Use

Condom use outcomes was assessed at both baseline and follow-up using the question “In the last three months, how often have you used condoms during anal sex?”, with the response options of *always*, *Most of the time*, *sometimes*, *rarely*, and *Never* for analyses, we dichotomized responses as always vs inconsistent (including all the other response options).

#### Qualitative Feedback

To evaluate acceptability, identify implementation challenges, and elicit suggestions to refine CHALO!, we collected field notes from (1) our weekly project meetings with the research team, (2) peer outreach staff during and at the end of study, and (3) two focus groups of CHALO! participants (n=6-8 per group) after intervention completion. Peer intervention staff also elicited feedback via email, WhatsApp, or Facebook Messenger from those not completing the follow-up assessment to evaluate reasons for survey noncompletion.

### Analyses

To determine feasibility, we assessed three process measures: (1) enrollment data (number of individuals completing the screening survey, the proportion eligible, and the proportion enrolling into the study); (2) retention (measured by a composite indicator consisting of WhatsApp and Facebook message viewed indicators, participant responses to reminder emails about completing the follow-up assessment, or completion of the follow-up assessment), and (3) completion rate (proportion of participants completing the follow-up assessment). We also assessed the relationship between completion of the follow-up assessment and baseline participant characteristics using the chi-square, Fisher exact, or *t* tests as appropriate.

To determine acceptability, we tabulated the Likert scale responses to questions about how much the group liked participating in CHALO! and used the chi-square test to examine differences between conditions. We thematically analyzed and coded the field notes and the brief open-ended responses on the follow-up survey based on three general categories: what was most liked, what was most disliked, and suggestions for improving the intervention. Two team members independently coded the responses, and discrepancies were resolved through discussion.

To determine early efficacy, we undertook several steps. We first described the sample using frequencies and means and examined potential differences in baseline characteristics and behaviors using the chi-square and *t* tests, as appropriate. To assess the intervention’s early impact, we examined potential changes in four outcomes: (1) composite of HIV testing plus intention to test, (2) HIV testing alone, (3) intention to test for HIV, and (4) consistent condom use. We first compared the two arms among those completing the postintervention assessment using chi-square tests. Next, we conducted a pooled pre-post analysis (ie, within-subjects and across time) for HIV testing, intention to test for HIV, and consistent condom use using the McNemar test.

## Results

### Participant Characteristics

**Figure 1:**
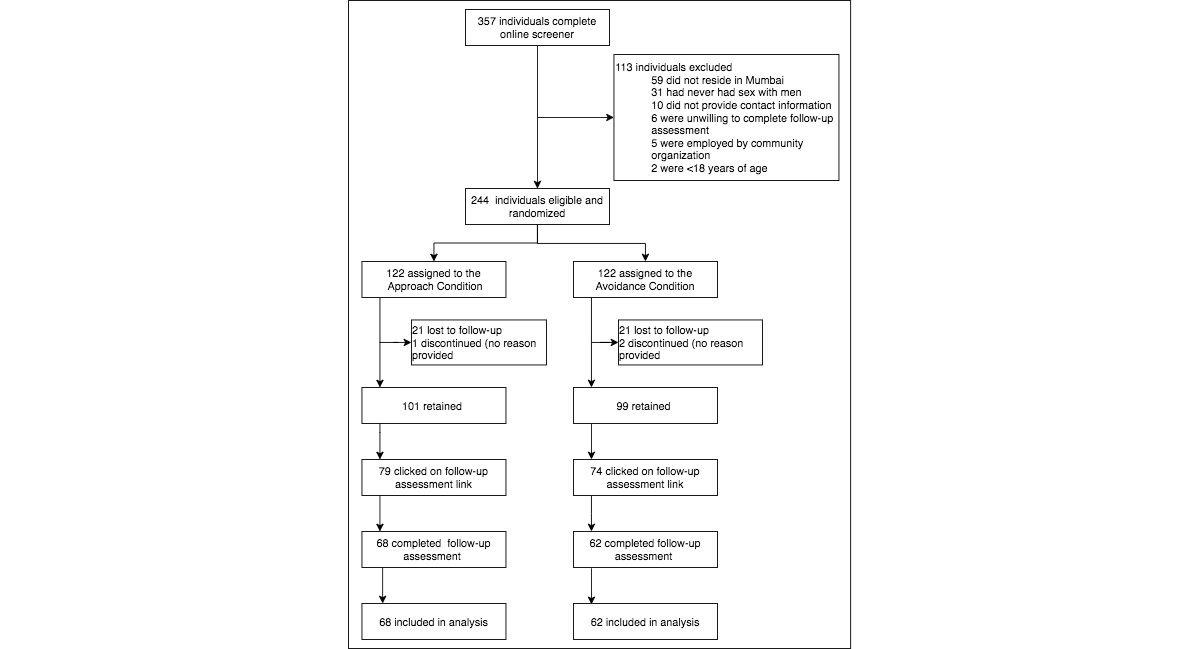
Consolidated Standards of Reporting Trials (CONSORT) flow diagram of CHALO! participants.

From February 2015 to March 2015, 982 individuals clicked through the advertised links, 357 (36.4%) individuals completed the online screening survey, of whom 244 (68.3%) were eligible; all eligible individuals enrolled (244/244, 100.0%) and were randomly assigned 1:1 to either the avoidance- or approach-framed conditions (122 in each arm; Figure 1).

Baseline participant characteristics by group assignment appear in Table 1. Overall, majority of participants were aged between 18 and 29 years (156/244, 63.9%), had monthly incomes of over INR 18,001 (155/244, 63.5%), and lived with other family members (151/244, 61.9%). Most individuals identified as gay or homosexual (175/244, 71.7%) or bisexual (63/244, 25.8%), nearly a quarter (56/244, 23.0%) were not out to anyone, and almost half (128/244, 52.5%) had never visited HST for any reason. Half of the participants (122/244, 50.0%) had a main male partner, and the overall sample reported a mean of 3.7 (SD 5) male sexual partners in the past 12 months. Half of all participants (128/244, 52.5%) chose to receive intervention messages via email, 35.7% (87/244) via WhatsApp, and 11.5% (28/244) through a private Facebook group. There were no significant differences at baseline between conditions with regard to any of the demographic characteristics, chosen mode for message delivery, or preferred language (Table 1).

**Table 1:** Baseline characteristics of participants in the CHALO! pilot.

Characteristics		Total (N=244)	Approach condition (n=122)	Avoidance condition (n=122)
**Age (years), n (%)^a^**				
	18-29	156 (63.9)	77 (63.1)	79 (64.8)
	30-41	64 (26.2)	33 (27)	31 (25.4)
	42 and above	24 (9.8)	11 (9)	12 (9.8)
**Monthly income (Indian Rupees**)**, n (%)^a^**				
	Rs 3000-9000 (approximately US $50-150)	55 (22.5)	28 (23)	27 (22.1)
	Rs 9001-18,000 (approximately US $150-300)	33 (13.5)	15 (12.3)	18 (14.8)
	> Rs 18,001 (> approximately US $300)	155 (63.5)	79 (64.8)	77 (63.1)
**Household members, n (%)^a^**				
	Alone	33 (13.5)	20 (16.4)	13 (10.6)
	Friends or nonrelative roommate	31 (12.7)	15 (12.3)	16 (13.1)
	Boyfriend, husband, male partner	9 (2.3)	2 (1.6)	7 (5.7)
	Joint family (eg, parents, siblings, relatives)	151 (61.9)	77 (63.1)	74 (60.7)
	Wife (female) or own children	14 (5.7)	7 (5.7)	7 (5.7)
**Preferred language, n (%)^a^**				
	English	225 (92.2)	113 (92.6)	112 (91.8)
	Hindi	19 (7.8)	9 (7.4)	10 (8.2)
**Sexual orientation, n (%)^a^**				
	Gay, homosexual, or queer	175 (71.7)	88 (72.1)	87 (71.3)
	Bisexual	63 (25.8)	31 (25.4)	32 (26.2)
	Straight or heterosexual	8 (3.3)	4 (3.3)	4 (3.3)
**Level of outness, n (%)^a^**				
	No one	56 (23.0)	27 (22.1)	29 (23.7)
	Some people	144 (59.0)	73 (59.8)	71 (58.2)
	Most people	44 (18.0)	22 (18)	22 (18)
Aware of Humsafar Trust (lesbian, gay, bisexual, transgender, and queer + community based organization), n (%)^a^		222 (91.0)	109 (89.3)	113 (92.6)
Have never visited Humsafar Trust, n (%)^a^		128 (52.5)	67 (54.9)	61 (50)
Has a main male partner, n (%)^a^		122 (50.0)	62 (50.8)	60 (49.2)
Number of male sexual partners in past year, mean (SD)		3.7 (5)	3.1 (3.2)	4.5 (6.4)
**Access to intervention contents, n (%)^a^**				
	Personal smartphone	136 (55.7)	66 (54.1)	70 (57.4)
	Home computer	73 (29.9)	39 (32)	34 (27.9)
	Work computer	20 (8.2)	9 (7.3)	11 (9)
	Friend’s computer	7 (2.9)	6 (4.9)	1 (0.8)
	Internet cafe	3 (1.2)	1 (0.8)	2 (1.6)
**Mode of message delivery, n (%)^a^**				
	Email	128 (52.5)	60 (49.1)	68 (55.7)
	Private Facebook group	28 (11.5)	15 (12.3)	13 (10.7)
	WhatsApp	87 (35.7)	46 (37.7)	41 (33.6)

^a^Percentages do not add up to 100% for all variables because of rounding.

### Feasibility and Retention

Overall, 82.0% (200/244) of the enrolled participants were retained through the end of the intervention (Figure 1). The postintervention assessment link was clicked on by 62.7% (153/244) participants and was completed by 53.3% (130/244) participants. There were no significant differences between conditions in the proportion of participants retained, accessing the follow-up assessment, and completing the follow-up assessment. There were also no significant differences in baseline demographic and behavioral characteristics among those retained or completing the follow-up assessment, except with regard to sexual orientation (Multimedia Appendix 1). Participants identifying as gay were more likely than those identifying as bisexual or straight to complete the follow-up assessment: 59.2% (103/174) for gay, 41% (26/63) for bisexual, and 13% (1/8) for straight; *P*<.01.

### Intervention Acceptability and Suggestions for Improvement

Of the 130 participants completing the postintervention assessment, 106 (81.5%) liked or strongly liked their experience in CHALO!, 17.9% (19/106) neither liked nor disliked their experience, and only 4.7% (5/106) disliked or strongly disliked their experience in CHALO! Content analysis of free-text responses about what participants liked most about CHALO! revealed the following themes: the intervention was useful and provided supportive information; messages were engaging or motivating; created a sense of community and acceptance; and made them feel good about helping their community by participating in the study. With regard to what participants least liked about CHALO!, individuals reported the survey was too long or redundant, reported feeling that messages were not frequent enough, or had comments pertaining to the graphical appearance of the messages. Suggestions for improvement included having a larger social media presence, continuing the messaging for a longer duration, expanding topics, and using audiovisual or interactive graphics (eg, video clips). Field notes and feedback from the peer outreach staff indicated that the most common reason for not completing the follow-up assessment was not finding the online Amazon India incentives useful.

### Potential Contamination

Of the participants completing the postintervention assessment (n=130), similar proportions of participants overall and in both conditions reported sharing the received digital messages with their friends (24% in both groups). In addition, 24% of individuals reported knowing someone else participating in CHALO! Only 6.1% (8/130) reported both knowing someone else in CHALO! and sharing the digital messages in general (which may or may not have been with the other CHALO participants).

### Preliminary Efficacy

#### HIV Testing

Table 2 shows the results of HIV testing outcomes for those who completed the follow-up survey (N=130). At baseline, 31.5% (41/130) of participants reported HIV testing in the past 6 months; at follow-up, 43.8% (57/130) of them reported having been tested (*P*=.04). When including those who reported intentions to test, this percentage increased from 44.6% (58/130) at baseline to 65.4% (85/130) at follow-up (*P*<.01). Finally, when examining intentions to test in the next month, among those without prior HIV testing, intentions increased from 32% (16/50) of the sample at baseline to 56% (28/50) of the sample at follow-up (*P*=.02).

At follow-up, fewer participants in approach vs avoidance reported being HIV tested in the past 6 months (26/68, 38% vs 31/68, 50%; *P*=.18) or intended to get an HIV test among those not tested in the past 6 months (47% vs 58%; *P*=.21), but these differences were not statistically significant (Table 2).

**Table 2:** HIV testing and condom use at baseline and follow-up.

Outcomes		Pre-post analysis				Approach			Avoidance			*P* value^a^
		N	Baseline, n (%)	Follow-up, n (%)	*P* value^b^	N	Baseline, n (%)	Follow-up, n (%)	N	Baseline, n (%)	Follow-up, n (%)	
**HIV testing**												
	HIV tested in the past 6 months (self-reported)	130	41 (31.5)	57 (43.8)	.04	68	22 (32)	26 (38)	62	19 (31)	31 (50)	.18
	HIV tested in the past 6 months (self-reported) or intent to test	130	58 (44.6)	85 (65.4)	<.01	68	32 (47)	41 (60)	62	26 (42)	44 (71)	.20
	Intent to HIV test among those not tested in the past 6 months	50	16 (32)	28 (56)	.02	28	10 (36)	15 (54)	22	6 (27)	13 (59)	.21
**Condom use^c^**												
	Always used condoms	76	47 (62)	46 (61)	.71	36	24 (64)	24 (64)	40	23 (58)	22 (55)	.41
	Condom use at the last anal sex encounter in the past 3 months	76	58 (76)	53 (70)	.29	36	28 (78)	29 (81)	40	27 (67)	23 (58)	.18

^a^Comparison between approach vs avoidance at follow-up.

^b^Comparison between baseline and follow-up.

^c^Condom use is among those reporting anal sex in past 3 months.

#### Condom Use

Among those having had anal sex in the past 3 months (n=76), the percentage of participants reporting always using a condom did not change overall (47/76, 62% at baseline vs 46/76, 61% at follow-up; *P=*.45), and there were no differences by arm (Table 2). There were also no significant differences in condom use at the last anal sex encounter between baseline and follow-up or between arms at follow-up (Table 2).

## Discussion

### Principal Findings

Using a community-based participatory research process, we developed and implemented an internet-based HIV prevention intervention for MSM in India. The findings from this pilot study showed that the CHALO! intervention delivery model was feasible to implement by a community-based organization and acceptable to participants, particularly those identifying as gay or homosexual. The intervention also demonstrated preliminary evidence for improving HIV testing and intention to test for HIV across both trial arms by self-report, with a greater increase in the avoidance-framed arm. However, the intervention had no impact on condom use.

To our knowledge, this is the first HIV-related intervention for MSM in India conducted exclusively online, using internet-based platforms to recruit, enroll, deliver a behavioral intervention, and follow-up participants longitudinally. We were able to reach and retain diverse MSM participants with respect to their sociodemographic characteristics, sexual identity, and level of outness. To our knowledge, almost all previous HIV-related intervention studies and service delivery programs in India have primarily relied on in-person approaches to initial outreach, and there exists only one published study that used mobile phones, but integrated the mobile device with in-person approaches for reaching individuals and delivering interventions [47]. A few other studies using diverse ICT-based platforms and intervention procedures have evaluated whether internet-based platforms can increase HIV testing among MSM in other low- and middle-income countries and, in general, have found overall positive effects, although none of them have been conducted in India or other South Asian countries and few are readily scalable with limited resources [27,48-52].

Our pilot study extends the literature by demonstrating the potential utility of a peer-delivered messaging intervention in a low-income country setting. Our findings also support the feasibility of implementing online interventions for MSM in India, with other recent data showing the ability to rapidly engage diverse Indian MSM online, including in rural areas [16]. Fully powered internet-based intervention studies are warranted to examine the impact of these online models with more objective measures of HIV testing and assessment of downstream outcomes of linkage to care (for both treatment and prevention). In addition, HIV prevention studies of longer duration of fully online interventions in India and other low-income settings are needed to understand long-term retention and program effectiveness. Unlike online interventions that rely on specific software platforms or require high technical expertise and resources, the CHALO! intervention model—including the rigorous community-based development process for message creation—may also serve as a model for future ICT-based interventions that are able to accommodate the constantly shifting sociotechnical landscape [53].

The use of message framing to inform online HIV-related messages has not previously been investigated anywhere; this study suggests that online educational and outreach interventions may need to consider the manner in which information is presented, depending on the health behavior being targeted. We found differences by study arm in the reports of HIV testing and intention to test, suggesting an influence of the message frame. Although both groups had significant increases in HIV testing outcomes from baseline to follow-up, we observed a greater increase among participants randomized to the avoidance-framed arm. This finding is consistent with the Prospect Theory [38], which posits that decision making is affected by framing effects. This study provides further support that framing effects may be dependent on whether the behavior is diagnostic (eg, HIV testing) or preventive (eg, condom use), which is consistent with previous studies [30,31,33,41]. In addition, framing effects on HIV testing behaviors may also be moderated by prior testing experiences; for example, avoidance- or negative-framed messages may work better for those who have never been tested for HIV but approach or positive-framed messages may work better for individuals previously tested for HIV [41,54], although we are unaware of any studies examining this issue. Although we were precluded from examining these types of potential effects stratified by prior testing history because of the small sample size, future studies could further examine these interactions to inform more tailored messaging interventions.

The CHALO! pilot did not have an impact on reported condom use behaviors. Increasing and sustaining condom use over time have been challenging across different contexts globally and interventions have had mixed findings [52,55,56]. This may be because of condom use being a complex behavior and one that requires the cooperation of the individual and their partner(s). Condom use is influenced by a variety of complex factors, including skills to use a condom, risk perception, influence of substances, community norms, desire for intimacy, stigma, and situational factors [57-61]. Thus, messaging alone is insufficient to address all these barriers. We hypothesize that CHALO! increased reported HIV testing behaviors but not condom use because obtaining an HIV test, in general, is an infrequent event compared with having sex. In addition, getting an HIV test may be more within the control of an individual’s decision making, whereas for using condoms they have to also rely on the preferences and decisions of the partner(s) [60,61]. Given the continued challenges in promoting and sustaining consistent condom use over time, other prevention modalities such as HIV pre-exposure prophylaxis (PrEP) and further development of online educational, outreach, and behavioral interventions are warranted. Although no single prevention modality will work for all, PrEP provides another highly effective option that is user centered. Notwithstanding other barriers to PrEP implementation, including awareness, access, cost, and provider-related obstacles, future research should examine the use of online interventions to promote PrEP adoption to MSM and other key populations in low- and middle-income countries.

### Limitations

This study should be interpreted in light of its limitations. First, our measures were self-reported, which may have introduced social desirability bias. However, given that we only observed changes in HIV testing and not for condom use, as well as the relatively anonymous nature of participant enrollment, social desirability may have played a limited role. Future studies with more objective measures are nevertheless needed. Second, this study recruited MSM online who reported living in Mumbai and thus may not be generalizable to online MSM elsewhere in India, particularly in settings that may not have MSM-sensitive physical services or a wide range of HIV testing sites available. Third, men who identified as bisexual and straight had low retention, indicating this pilot intervention likely had minimal impact on these groups who may be at higher risk for HIV [62,63]. Future internet-based interventions for HIV prevention may benefit from taking into account sexual identity and tailoring contents specific to bisexual- and straight-identifying MSM. Finally, there could have been potential contamination between the study arms, given that a quarter of participants reported sharing the digital messages and a quarter knew of others participating in the study. Given the nature of online interventions with commonly used platforms, some degree of contamination is inevitable, and further research is needed to understand how best to measure and minimize contamination in online studies. Studies with larger samples are also needed to examine the impact of contamination on outcomes. However, a strength of social media and online interventions is their ability to rapidly diffuse information, and thus research is needed to understand how interventions could leverage the possibility of contamination as a strength rather than a limitation.

### Conclusions

As one of the first studies of an online HIV intervention in India, this pilot study demonstrated preliminary efficacy for increasing self-reported HIV testing in an urban sample of MSM reached online, with the potential for wide national reach and high feasibility and acceptability. Given the continued structural challenges in engaging MSM in public health efforts (eg, stigma at various levels and lack of MSM-affirmative health care), changes in how MSM socialize and find partners and the suboptimal HIV testing rates—particularly in stigmatized settings—ICT-based intervention delivery models present new opportunities to engage MSM with or at high risk of HIV into care and prevention. Our findings signal the need for efficacy testing of this type of scalable intervention in a fully powered trial with objective measures of actual HIV testing and assessing its impact on downstream outcomes of linkage to care and prevention services.

## Appendix

Multimedia Appendix 1 Baseline demographic characteristics of intervention participants completing and not completing the postassessment survey (N=244).

Multimedia Appendix 2 CONSORT-EHEALTH checklist (V 1.6.1).

## Abbreviations

HST: Humsafar Trust
ICT: internet-based communication technology
IMB: information-motivational-behavioral
MSM: men who have sex with men
NIH: National Institutes of Health
PrEP: pre-exposure prophylaxis
STI: sexually transmitted infection
