# Supplementary material for: An Internet-Based, Peer-Delivered Messaging Intervention for HIV Testing and Condom Use Among Men Who Have Sex With Men in India (CHALO!): Pilot Randomized Comparative Trial
Source: JMIR Public Health Surveill. 2020 Apr 16;6(2):e16494. doi: 10.2196/16494 (PMC7193444; doi:10.2196/16494)
Supplement: Multimedia Appendix 1 [file publichealth_v6i2e16494_app1.docx]

|  | Total  N (%) | Completers  n (%) | Non-completers  n (%) | P |
| --- | --- | --- | --- | --- |
| Total | 244 | 130 | 114 |  |
| Age |  |  |  | *n.s.* |
| 18-29 | 156 (64) | 79 (61) | 77 (68) |  |
| 30-41 | 64 (26) | 40 (31) | 24 (21) |  |
| 42 and above | 24 (10) | 11 (9) | 13 (11) |  |
| Sexual Orientation |  |  |  | < 0.01 |
| Gay | 174 (71) | 103 (79) | 71 (62) |  |
| Bisexual | 63 (26) | 26 (20) | 37 (33) |  |
| Straight | 7 (3) | 1 (1) | 6 (5) |  |
| Level of Outness |  |  |  | *n.s.* |
| Out to few | 205 (84) | 107 (82) | 98 (87) |  |
| Mostly out | 38 (16) | 23 (18) | 15 (13) |  |
| Income |  |  |  | *n.s.* |
| <3,000-9,000 | 56 (23) | 25 (19) | 31 (27) |  |
| 9,001-15,000 | 19 (8) | 13 (10) | 6 (5) |  |
| > 15,001 | 169 (69) | 92 (71) | 77 (68) |  |
| Main Partner |  |  |  | *n.s.* |
| Yes | 140 (57) | 80 (62) | 60 (53) |  |
| No | 104 (43) | 50 (39) | 54 (47) |  |
| Number of male partners in past year Mean (SD) | 4.1 (7.9) | 3.8 (5.0) | 4.6 (10.3) | *n.s.* |
