# Supplementary material for: An Internet-Based, Peer-Delivered Messaging Intervention for HIV Testing and Condom Use Among Men Who Have Sex With Men in India (CHALO!): Pilot Randomized Comparative Trial
Source: JMIR Public Health Surveill. 2020 Apr 16;6(2):e16494. doi: 10.2196/16494 (PMC7193444; doi:10.2196/16494)
Supplement: Multimedia Appendix 2 [file publichealth_v6i2e16494_app2.pdf]

CONSORT-EHEALTH (V 1.6.1) - Submission/Publication Form

The CONSORT-EHEALTH checklist is intended for authors of randomized trials evaluating web-based and Internet-based applications/interventions, including mobile interventions, electronic games (incl multiplayer games), social media, certain telehealth applications, and other interactive and/or networked electronic applications. Some of the items (e.g. all subitems under item 5 - description of the intervention) may also be applicable for other study designs.

The goal of the CONSORT EHEALTH checklist and guideline is to be
a) a guide for reporting for authors of RCTs,
b) to form a basis for appraisal of an ehealth trial (in terms of validity)

CONSORT-EHEALTH items/subitems are MANDATORY reporting items for studies published in the Journal of Medical Internet Research and other journals / scientific societies endorsing the checklist.

Items numbered 1., 2., 3., 4a., 4b etc are original CONSORT or CONSORT-NPT (non-pharmacologic treatment) items.
Items with Roman numerals (i., ii, iii, iv etc.) are CONSORT-EHEALTH extensions/clarifications.

As the CONSORT-EHEALTH checklist is still considered in a formative stage, we would ask that you also RATE ON A SCALE OF 1-5 how important/useful you feel each item is FOR THE PURPOSE OF THE CHECKLIST and reporting guideline (optional).

Mandatory reporting items are marked with a red *.
In the textboxes, either copy & paste the relevant sections from your manuscript into this form - please include any quotes from your manuscript in QUOTATION MARKS, or answer directly by providing additional information not in the manuscript, or elaborating on why the item was not relevant for this study.

YOUR ANSWERS WILL BE PUBLISHED AS A SUPPLEMENTARY FILE TO YOUR PUBLICATION IN JMIR AND ARE CONSIDERED PART OF YOUR PUBLICATION (IF ACCEPTED).
Please fill in these questions diligently. Information will not be copyedited, so please use proper spelling and grammar, use correct capitalization, and avoid abbreviations.

DO NOT FORGET TO SAVE AS PDF _AND_ CLICK THE SUBMIT BUTTON SO YOUR ANSWERS ARE IN OUR DATABASE !!!

Citation Suggestion (if you append the pdf as Appendix we suggest to cite this paper in the caption):
Eysenbach G, CONSORT-EHEALTH Group
CONSORT-EHEALTH: Improving and Standardizing Evaluation Reports of Web-based and Mobile Health Interventions
J Med Internet Res 2011;13(4):e126
URL: http://www.jmir.org/2011/4/e126/
doi: 10.2196/jmir.1923
PMID: 22209829

* Required

Your name *

First Last

Viraj Patel

Primary Affiliation (short), City, Country *

University of Toronto, Toronto, Canada

Division of General Internal Medicine, Depa

Your e-mail address *

abc@gmail.com

vpatel@montefiore.org

Title of your manuscript *

Provide the (draft) title of your manuscript.

An Internet-Based, Peer-Delivered Messaging Intervention to Increase Self-Reported HIV Testing Behaviors Among Men Who Have Sex With Men in India (CHALO!): Pilot Randomized Controlled Trial

Name of your App/Software/Intervention *

If there is a short and a long/alternate name, write the short name first and add the long name in brackets.

CHALO!

Evaluated Version (if any)

e.g. "V1", "Release 2017-03-01", "Version 2.0.27913"

Your answer

Language(s) *

What language is the intervention/app in? If multiple languages are available, separate by comma (e.g. "English, French")

English

URL of your Intervention Website or App

e.g. a direct link to the mobile app on app in appstore (itunes, Google Play), or URL of the website. If the intervention is a DVD or hardware, you can also link to an Amazon page.

Your answer

URL of an image/screenshot (optional)

Your answer

Accessibility *

Can an enduser access the intervention presently?

- ○ access is free and open
- ○ access only for special usergroups, not open
- ○ access is open to everyone, but requires payment/subscription/in-app purchases
- ◉ app/intervention no longer accessible
- ○ Other:

Primary Medical Indication/Disease/Condition *

e.g. "Stress", "Diabetes", or define the target group in brackets after the condition, e.g. "Autism (Parents of children with)", "Alzheimers (Informal Caregivers of)"

HIV Testing and Condom use (MSM)

Primary Outcomes measured in trial *

comma-separated list of primary outcomes reported in the trial

Feasibility; Acceptability; HIV Testing; Cons

Secondary/other outcomes

Are there any other outcomes the intervention is expected to affect?

Intention to get an HIV test

Recommended "Dose" *

What do the instructions for users say on how often the app should be used?

- ( ) Approximately Daily
- ( ) Approximately Weekly
- ( ) Approximately Monthly
- ( ) Approximately Yearly
- ( ) "as needed"
- (•) Other: 2-3 times a week over 12 weeks

Approx. Percentage of Users (starters) still using the app as recommended after 3 months *

- ○ unknown / not evaluated
- ○ 0-10%
- ○ 11-20%
- ○ 21-30%
- ○ 31-40%
- ○ 41-50%
- ○ 51-60%
- ○ 61-70%
- ○ 71%-80%
- ◉ 81-90%
- ○ 91-100%
- ○ Other:

Overall, was the app/intervention effective? *

- ( ) yes: all primary outcomes were significantly better in intervention group vs control
- (•) partly: SOME primary outcomes were significantly better in intervention group vs control
- ( ) no statistically significant difference between control and intervention
- ( ) potentially harmful: control was significantly better than intervention in one or more outcomes
- ( ) inconclusive: more research is needed
- ( ) Other:

Article Preparation Status/Stage *

At which stage in your article preparation are you currently (at the time you fill in this form)

- ( ) not submitted yet - in early draft status
- ( ) not submitted yet - in late draft status, just before submission
- ( ) submitted to a journal but not reviewed yet
- ( ) submitted to a journal and after receiving initial reviewer comments
- (•) submitted to a journal and accepted, but not published yet
- ( ) published
- ( ) Other:

Journal *

If you already know where you will submit this paper (or if it is already submitted), please provide the journal name (if it is not JMIR, provide the journal name under "other")

- ○ not submitted yet / unclear where I will submit this
- ○ Journal of Medical Internet Research (JMIR)
- ◉ JMIR mHealth and UHealth
- ○ JMIR Serious Games
- ○ JMIR Mental Health
- ○ JMIR Public Health
- ○ JMIR Formative Research
- ○ Other JMIR sister journal
- ○ Other:

Is this a full powered effectiveness trial or a pilot/feasibility trial? *

- ◉ Pilot/feasibility
- ○ Fully powered

Manuscript tracking number *

If this is a JMIR submission, please provide the manuscript tracking number under "other" (The ms tracking number can be found in the submission acknowledgement email, or when you login as author in JMIR. If the paper is already published in JMIR, then the ms tracking number is the four-digit number at the end of the DOI, to be found at the bottom of each published article in JMIR)

- ○ no ms number (yet) / not (yet) submitted to / published in JMIR
- ● Other: ms#16494

TITLE AND ABSTRACT

1a) TITLE: Identification as a randomized trial in the title

1a) Does your paper address CONSORT item 1a? *

I.e does the title contain the phrase "Randomized Controlled Trial"? (if not, explain the reason under "other")

- ● yes
- ○ Other:

1a-i) Identify the mode of delivery in the title

Identify the mode of delivery. Preferably use “web-based” and/or “mobile” and/or “electronic game” in the title. Avoid ambiguous terms like “online”, “virtual”, “interactive”. Use “Internet-based” only if Intervention includes non-web-based Internet components (e.g. email), use “computer-based” or “electronic” only if offline products are used. Use “virtual” only in the context of “virtual reality” (3-D worlds). Use “online” only in the context of “online support groups”. Complement or substitute product names with broader terms for the class of products (such as “mobile” or “smart phone” instead of “iphone”), especially if the application runs on different platforms.

	1	2	3	4	5	
subitem not at all important	○	○	○	○	◉	essential

Does your paper address subitem 1a-i? *

Copy and paste relevant sections from manuscript title (include quotes in quotation marks "like this" to indicate direct quotes from your manuscript), or elaborate on this item by providing additional information not in the ms, or briefly explain why the item is not applicable/relevant for your study

"Leveraging Internet based communication tools (e.g, messaging apps, SMS, email) may be an effective avenue for delivery of HIV prevention messages to men who have sex with men (MSM) in India, but there are limited models for such online

1a-ii) Non-web-based components or important co-interventions in title

Mention non-web-based components or important co-interventions in title, if any (e.g., “with telephone support”).

	1	2	3	4	5	
subitem not at all important	◉	○	○	○	○	essential

Does your paper address subitem 1a-ii?

Copy and paste relevant sections from manuscript title (include quotes in quotation marks "like this" to indicate direct quotes from your manuscript), or elaborate on this item by providing additional information not in the ms, or briefly explain why the item is not applicable/relevant for your study

Your answer

1a-iii) Primary condition or target group in the title

Mention primary condition or target group in the title, if any (e.g., "for children with Type I Diabetes") Example: A Web-based and Mobile Intervention with Telephone Support for Children with Type I Diabetes: Randomized Controlled Trial

	1	2	3	4	5	
subitem not at all important	○	○	○	○	◉	essential

Does your paper address subitem 1a-iii? *

Copy and paste relevant sections from manuscript title (include quotes in quotation marks "like this" to indicate direct quotes from your manuscript), or elaborate on this item by providing additional information not in the ms, or briefly explain why the item is not applicable/relevant for your study

"Globally and in India, HIV disproportionately affects men who have sex with men (MSM), who are designated a key priority population by the Indian health ministry for targeted HIV prevention interventions "

1b) ABSTRACT: Structured summary of trial design, methods, results, and conclusions

NPT extension: Description of experimental treatment, comparator, care providers, centers, and blinding status.

1b-i) Key features/functionalities/components of the intervention and comparator in the METHODS section of the ABSTRACT

Mention key features/functionalities/components of the intervention and comparator in the abstract. If possible, also mention theories and principles used for designing the site. Keep in mind the needs of systematic reviewers and indexers by including important synonyms. (Note: Only report in the abstract what the main paper is reporting. If this information is missing from the main body of text, consider adding it)

	1	2	3	4	5	
subitem not at all important	○	○	○	◉	○	essential

Does your paper address subitem 1b-i? *

Copy and paste relevant sections from the manuscript abstract (include quotes in quotation marks "like this" to indicate direct quotes from your manuscript), or elaborate on this item by providing additional information not in the ms, or briefly explain why the item is not applicable/relevant for your study

"Eligible men (18 years or older, sexually active with other men, and self-reported HIV-negative or unknown status) were randomized to receive educational and motivational messages framed as either: Approach (i.e., a desirable outcome to be achieved) or Avoidance (an undesirable outcome to be avoided) over 12 weeks via Internet-based messaging platforms"

1b-ii) Level of human involvement in the METHODS section of the ABSTRACT

Clarify the level of human involvement in the abstract, e.g., use phrases like “fully automated” vs. “therapist/nurse/care provider/physician-assisted” (mention number and expertise of providers involved, if any). (Note: Only report in the abstract what the main paper is reporting. If this information is missing from the main body of text, consider adding it)

	1	2	3	4	5	
subitem not at all important	○	○	◉	○	○	essential

Does your paper address subitem 1b-ii?

Copy and paste relevant sections from the manuscript abstract (include quotes in quotation marks "like this" to indicate direct quotes from your manuscript), or elaborate on this item by providing additional information not in the ms, or briefly explain why the item is not applicable/relevant for your study

"receive educational and motivational messages framed as either approach (ie, a desirable outcome to be achieved) or avoidance (an undesirable outcome to be avoided) over 12 weeks via internet-based messaging platforms."

1b-iii) Open vs. closed, web-based (self-assessment) vs. face-to-face assessments in the METHODS section of the ABSTRACT

Mention how participants were recruited (online vs. offline), e.g., from an open access website or from a clinic or a closed online user group (closed usergroup trial), and clarify if this was a purely web-based trial, or there were face-to-face components (as part of the intervention or for assessment). Clearly say if outcomes were self-assessed through questionnaires (as common in web-based trials). Note: In traditional offline trials, an open trial (open-label trial) is a type of clinical trial in which both the researchers and participants know which treatment is being administered. To avoid confusion, use “blinded” or “unblinded” to indicated the level of blinding instead of “open”, as “open” in web-based trials usually refers to “open access” (i.e. participants can self-enrol). (Note: Only report in the abstract what the main paper is reporting. If this information is missing from the main body of text, consider adding it)

	1	2	3	4	5	
subitem not at all important	○	○	○	●	○	essential

Does your paper address subitem 1b-iii?

Copy and paste relevant sections from the manuscript abstract (include quotes in quotation marks "like this" to indicate direct quotes from your manuscript), or elaborate on this item by providing additional information not in the ms, or briefly explain why the item is not applicable/relevant for your study

"receive educational and motivational messages framed as either approach (ie, a desirable outcome to be achieved) or avoidance (an undesirable outcome to be avoided) over 12 weeks via internet-based messaging platforms."

1b-iv) RESULTS section in abstract must contain use data

Report number of participants enrolled/assessed in each group, the use/uptake of the intervention (e.g., attrition/adherence metrics, use over time, number of logins etc.), in addition to primary/secondary outcomes. (Note: Only report in the abstract what the main paper is reporting. If this information is missing from the main body of text, consider adding it)

	1	2	3	4	5	
subitem not at all important	○	○	○	◉	○	essential

Does your paper address subitem 1b-iv?

Copy and paste relevant sections from the manuscript abstract (include quotes in quotation marks "like this" to indicate direct quotes from your manuscript), or elaborate on this item by providing additional information not in the ms, or briefly explain why the item is not applicable/relevant for your study

"Over 82% of participants (n=200) were retained (ie, viewed final messages), and 53% (n=130) completed the follow-up survey. Of those completing the follow-up survey, 82% liked or strongly liked participating in CHALO!. The results showed a significant increase in self-reported HIV-testing in the past 6 months from baseline to follow-up (32% to 44%; P<.05). When including those who reported intentions to test, this percentage increased from 45% at baseline to 65% at follow-up (P<.01). When examining intentions to test among those without prior HIV-testing, intentions increased from 32% of the sample at baseline, to 56% of the sample at follow-up (P<.05) Condom use during anal sex did not significantly change from baseline to follow-up. HIV-testing and condom use did not significantly differ between Approach

1b-v) CONCLUSIONS/DISCUSSION in abstract for negative trials

Conclusions/Discussions in abstract for negative trials: Discuss the primary outcome - if the trial is negative (primary outcome not changed), and the intervention was not used, discuss whether negative results are attributable to lack of uptake and discuss reasons. (Note: Only report in the abstract what the main paper is reporting. If this information is missing from the main body of text, consider adding it)

	1	2	3	4	5	
subitem not at all important	○	○	◉	○	○	essential

Does your paper address subitem 1b-v?

Copy and paste relevant sections from the manuscript abstract (include quotes in quotation marks "like this" to indicate direct quotes from your manuscript), or elaborate on this item by providing additional information not in the ms, or briefly explain why the item is not applicable/relevant for your study

Condom use during anal sex did not significantly change from baseline to follow-up. HIV-testing and condom use did not significantly differ between Approach and Avoidance conditions at follow-up.

INTRODUCTION

2a) In INTRODUCTION: Scientific background and explanation of rationale

2a-i) Problem and the type of system/solution

Describe the problem and the type of system/solution that is object of the study: intended as stand-alone intervention vs. incorporated in broader health care program? Intended for a particular patient population? Goals of the intervention, e.g., being more cost-effective to other interventions, replace or complement other solutions? (Note: Details about the intervention are provided in "Methods" under 5)

	1	2	3	4	5	
subitem not at all important	○	○	○	○	◉	essential

Does your paper address subitem 2a-i? *

Copy and paste relevant sections from the manuscript (include quotes in quotation marks "like this" to indicate direct quotes from your manuscript), or elaborate on this item by providing additional information not in the ms, or briefly explain why the item is not applicable/relevant for your study

"India has the 3rd largest population of people living with HIV globally, with an estimated 2.1 million infected persons, and more than 80,000 new infections occurring annually [1]. Globally and in India, HIV disproportionately affects men who have sex with men (MSM), who are designated a key priority population by the Indian health ministry for targeted HIV prevention interventions [2]. HIV prevalence among MSM in India is 10-15 times higher than in the general population (4.3% vs 0.3%) [1], and although MSM are conservatively estimated to make-up less than 2% of the population, they comprise over 20% of HIV infected individuals [1, 2]. Thus, current interventions for Indian MSM have been limited in their reach and impact [3], in part due to the social stigma associated with same-sex behaviors and marginalization [4, 5]. The cultural emphasis on heterosexual marriage and traditional family structure, coupled with the constant fear of one's sexuality being 'outed [6-9],' drive many MSM 'underground' and out of the purview of various HIV interventions currently being implemented. To reduce the burden of HIV among Indian MSM, rapid development and wide-scale dissemination of interventions

2a-ii) Scientific background, rationale: What is known about the (type of) system

Scientific background, rationale: What is known about the (type of) system that is the object of the study (be sure to discuss the use of similar systems for other conditions/diagnoses, if appropiate), motivation for the study, i.e. what are the reasons for and what is the context for this specific study, from which stakeholder viewpoint is the study performed, potential impact of findings [2]. Briefly justify the choice of the comparator.

	1	2	3	4	5	
subitem not at all important	○	○	○	◉	○	essential

Does your paper address subitem 2a-ii? *

Copy and paste relevant sections from the manuscript (include quotes in quotation marks "like this" to indicate direct quotes from your manuscript), or elaborate on this item by providing additional information not in the ms, or briefly explain why the item is not applicable/relevant for your study

"International organizations, including India's National AIDS Control Organization (NACO), recognize the public health potential of ICT, and have called for development and implementation of ICT-based HIV prevention strategies [17]. Besides evidence of their being acceptable to MSM, ICT also offer considerable scalability and efficiency of wide reach with high impact potential, even with relatively low intensity interventions [18, 19]. Technology-based, peer-led approaches could be used to enhance efforts by community based and other organizations for dissemination of health messages and service availability [20]. Thus, rather than an alternative medium for implementation of existing interventions designed for face-to-face contact, social media may be a 'game changer' to engage MSM in India [21]. A meta-analysis found that social media interventions were effective in increasing HIV-testing, however none of the studies were conducted in a low-income country [22]. Two recent systematic reviews describing internet based interventions for HIV care continuum found diverse models targeting HIV-testing and prevention; however, most (over 85%) were in well-resource settings.[23, 24] Few effective, scalable, and low-cost ICT-based interventions targeting HIV-testing and prevention exist for low income countries [25-28] and no published data are available on the effectiveness of ICT based approaches in India or other South Asian countries. In addition to the paucity of data about Internet based interventions in low-income settings for any population, there is little empirical data to guide health communication, i.e., messaging, for online dissemination to increase HIV-testing and condom use. Two messaging approaches, often called "frames" are widely used in health communication. The first, called "approach" or "gain framed, highlights the benefits of engaging in a specific health behavior. The second, called, "avoidance" or "loss-framed," focuses on negative consequences. Metanalytic reviews have indicated that gain-framed messaging is more effective in promoting prevention behavior, but loss-framed messaging may be more effective in promoting screening or illness detection behavior [29-31]. However, it is unclear which framing strategy is most effective when promoting a comprehensive approach to HIV prevention that includes promoting both HIV-testing and condom use. Prior research from a high-income country (United States) has found mixed-results with regards to condom use intentions [32, 33] and HIV-testing behaviors,[34]. However, to our knowledge, no published data exist with regards to framing effects of health messages for HIV-

2b) In INTRODUCTION: Specific objectives or hypotheses

Does your paper address CONSORT subitem 2b? *

Copy and paste relevant sections from the manuscript (include quotes in quotation marks "like this" to indicate direct quotes from your manuscript), or elaborate on this item by providing additional information not in the ms, or briefly explain why the item is not applicable/relevant for your study

"To help close the gap in the use of ICT for public health purposes in India and address the high HIV-prevention needs of Indian MSM, the CHALO! ("Let's Go!") pilot study, developed and tested the feasibility, acceptability, and preliminary impact of an ICT-based HIV prevention intervention to increase HIV-testing and consistent condom use among MSM reached on internet-based social and dating platforms in Mumbai, India. " and " Our central hypothesis was that a peer-delivered ICT-based behavioral intervention can efficiently identify and reach sexually active Indian MSM, enroll them into an exclusively online study, motivate them to seek in-person health services (i.e., HIV-testing), and modify health promotion behaviors (increase

METHODS

3a) Description of trial design (such as parallel, factorial) including allocation ratio

Does your paper address CONSORT subitem 3a? *

Copy and paste relevant sections from the manuscript (include quotes in quotation marks "like this" to indicate direct quotes from your manuscript), or elaborate on this item by providing additional information not in the ms, or briefly explain why the item is not applicable/relevant for your study

"CHALO! was a two-arm parallel randomized (1:1 randomization) comparative effectiveness trial comparing two message framing strategies (Avoidance- and Approach-framed messages) to promote HIV-testing and consistent condom use. "

3b) Important changes to methods after trial commencement (such as eligibility criteria), with reasons

Does your paper address CONSORT subitem 3b? *

Copy and paste relevant sections from the manuscript (include quotes in quotation marks "like this" to indicate direct quotes from your manuscript), or elaborate on this item by providing additional information not in the ms, or briefly explain why the item is not applicable/relevant for your study

There were no important changes to the methods after the trial commencement.

3b-i) Bug fixes, Downtimes, Content Changes

Bug fixes, Downtimes, Content Changes: ehealth systems are often dynamic systems. A description of changes to methods therefore also includes important changes made on the intervention or comparator during the trial (e.g., major bug fixes or changes in the functionality or content) (5-iii) and other “unexpected events” that may have influenced study design such as staff changes, system failures/downtimes, etc. [2].

	1	2	3	4	5	
subitem not at all important	(●)	( )	( )	( )	( )	essential

Does your paper address subitem 3b-i?

Copy and paste relevant sections from the manuscript (include quotes in quotation marks "like this" to indicate direct quotes from your manuscript), or elaborate on this item by providing additional information not in the ms, or briefly explain why the item is not applicable/relevant for your study

There are no bug fixes, downtimes, or content changes to report

4a) Eligibility criteria for participants

Does your paper address CONSORT subitem 4a? *

Copy and paste relevant sections from the manuscript (include quotes in quotation marks "like this" to indicate direct quotes from your manuscript), or elaborate on this item by providing additional information not in the ms, or briefly explain why the item is not applicable/relevant for your study

"Eligible individuals were aged at least 18 years, identified as male, reported anal sex with another male partner in the past 2 years, lived in Mumbai, were fluent in either English or Hindi, self-reported being HIV-negative, or unaware of their status (ie, never tested or never received results), and provided a valid contact (email, mobile phone number, or Facebook ID – validated by a response to a confirmation message). Individuals were excluded if they reported being a staff member or any type of outreach worker for HST. Participants were screened into the study using an

4a-i) Computer / Internet literacy

Computer / Internet literacy is often an implicit "de facto" eligibility criterion - this should be explicitly clarified.

	1	2	3	4	5	
subitem not at all important	○	○	◉	○	○	essential

Does your paper address subitem 4a-i?

Copy and paste relevant sections from the manuscript (include quotes in quotation marks "like this" to indicate direct quotes from your manuscript), or elaborate on this item by providing additional information not in the ms, or briefly explain why the item is not applicable/relevant for your study

"provided a valid contact (email, mobile phone number, or Facebook ID – validated by a response to a confirmation message). Individuals "

4a-ii) Open vs. closed, web-based vs. face-to-face assessments:

Open vs. closed, web-based vs. face-to-face assessments: Mention how participants were recruited (online vs. offline), e.g., from an open access website or from a clinic, and clarify if this was a purely web-based trial, or there were face-to-face components (as part of the intervention or for assessment), i.e., to what degree got the study team to know the participant. In online-only trials, clarify if participants were quasi-anonymous and whether having multiple identities was possible or whether technical or logistical measures (e.g., cookies, email confirmation, phone calls) were used to detect/prevent these.

	1	2	3	4	5	
subitem not at all important	○	○	○	◉	○	essential

Does your paper address subitem 4a-ii? *

Copy and paste relevant sections from the manuscript (include quotes in quotation marks "like this" to indicate direct quotes from your manuscript), or elaborate on this item by providing additional information not in the ms, or briefly explain why the item is not applicable/relevant for your study

"from March to June, 2015, the four peer-outreach workers (two per arm) sent a standardized introductory message via the participant’s chosen communication modality (ie, email, WhatsApp, or private Facebook group), followed by intervention messages two or three times per week for 12 weeks. Each set of 15 messages was sent out twice over the 12 weeks to help ensure that participants viewed them and reinforce the information contained within the messages. Thus, after the first set of 15 messages were sent out, another round of the same 15 messages was sent again. Messages for all participants were sent by the peer-outreach worker on the same days and times each week. Participants were also able to communicate with their assigned peer outreach worker for any reason via multiple modalities (text message, email, Facebook Messenger, or phone call), but only if the participant initiated contact. "

4a-iii) Information giving during recruitment

Information given during recruitment. Specify how participants were briefed for recruitment and in the informed consent procedures (e.g., publish the informed consent documentation as appendix, see also item X26), as this information may have an effect on user self-selection, user expectation and may also bias results.

	1	2	3	4	5	
subitem not at all important	○	○	○	◉	○	essential

Does your paper address subitem 4a-iii?

Copy and paste relevant sections from the manuscript (include quotes in quotation marks "like this" to indicate direct quotes from your manuscript), or elaborate on this item by providing additional information not in the ms, or briefly explain why the item is not applicable/relevant for your study

"First, from February to March 2015, recruitment advertisements were disseminated on a popular MSM specific dating website, a geosocial networking mobile app, and on HST operated/subscribed Facebook pages. Potential participants clicked through the ads to complete an online consent and eligibility screener; if eligible, they automatically continued on to the baseline survey."

4b) Settings and locations where the data were collected

Does your paper address CONSORT subitem 4b? *

Copy and paste relevant sections from the manuscript (include quotes in quotation marks "like this" to indicate direct quotes from your manuscript), or elaborate on this item by providing additional information not in the ms, or briefly explain why the item is not applicable/relevant for your study

"The study took place online, between February and June 2015, targeting MSM living in Mumbai – India's largest city with a population of over 18 million, and a city with one of the highest HIV burdens in India. Mumbai accounted for over 19,000 new HIV diagnoses in 2016-2017 [17, 35], with the prevalence of HIV among MSM estimated to be 7%. At the time of the study, Mumbai had high Internet connectivity with an abundance of free or low-cost Wi-Fi spots, low-cost Internet cafes, and mobile service providers offering Internet/data plans for smartphones at a relatively low cost. For this pilot, recruited participants from two of India's most used MSM specific dating sites (which have now become the most commonly used avenues for MSM meet in urban India [36, 37]) and from HST-operated Facebook pages. HST has three drop-in centers and a central office in the three major subdivisions of Mumbai, where HIV and STI testing, sexual health and psycho-social counseling, and linkage-to-care services are available. At the time of the study, HST was the only established community organization providing such services to MSM. "

4b-i) Report if outcomes were (self-)assessed through online questionnaires

Clearly report if outcomes were (self-)assessed through online questionnaires (as common in web-based trials) or otherwise.

	1	2	3	4	5	
subitem not at all important	○	○	○	◉	○	essential

Does your paper address subitem 4b-i? *

Copy and paste relevant sections from the manuscript (include quotes in quotation marks "like this" to indicate direct quotes from your manuscript), or elaborate on this item by providing additional information not in the ms, or briefly explain why the item is not applicable/relevant for your study

"Potential participants clicked through the ads to complete an online consent and eligibility screener; if eligible, they automatically continued on to the baseline survey. " and". "Participants then received a final intervention message and a personal link to the follow-up survey. "

4b-ii) Report how institutional affiliations are displayed

Report how institutional affiliations are displayed to potential participants [on ehealth media], as affiliations with prestigious hospitals or universities may affect volunteer rates, use, and reactions with regards to an intervention.(Not a required item – describe only if this may bias results)

	1	2	3	4	5	
subitem not at all important	○	○	○	○	○	essential

Does your paper address subitem 4b-ii?

Copy and paste relevant sections from the manuscript (include quotes in quotation marks "like this" to indicate direct quotes from your manuscript), or elaborate on this item by providing additional information not in the ms, or briefly explain why the item is not applicable/relevant for your study

Your answer

5) The interventions for each group with sufficient details to allow replication, including how and when they were actually administered

5-i) Mention names, credential, affiliations of the developers, sponsors, and owners

Mention names, credential, affiliations of the developers, sponsors, and owners [6] (if authors/evaluators are owners or developer of the software, this needs to be declared in a "Conflict of interest" section or mentioned elsewhere in the manuscript).

	1	2	3	4	5	
subitem not at all important	○	○	○	●	○	essential

Does your paper address subitem 5-i?

Copy and paste relevant sections from the manuscript (include quotes in quotation marks "like this" to indicate direct quotes from your manuscript), or elaborate on this item by providing additional information not in the ms, or briefly explain why the item is not applicable/relevant for your study

"We used a participatory process with an interdisciplinary team at HST to develop all components of the intervention in an iterative process over a three-month period. The core team members at HST consisted of eight individuals: two community-based researchers, two HIV-testing/counseling staff members, an HIV-positive peer patient navigator, and three peer outreach workers experienced in using MSM dating websites and apps for outreach to MSM in Mumbai. This intervention development team informed all aspects of the study including study design, participant eligibility, recruitment and retention, study measures, intervention implementation, and

5-ii) Describe the history/development process

Describe the history/development process of the application and previous formative evaluations (e.g., focus groups, usability testing), as these will have an impact on adoption/use rates and help with interpreting results.

	1	2	3	4	5	
subitem not at all important	○	○	○	●	○	essential

Does your paper address subitem 5-ii?

Copy and paste relevant sections from the manuscript (include quotes in quotation marks "like this" to indicate direct quotes from your manuscript), or elaborate on this item by providing additional information not in the ms, or briefly explain why the item is not applicable/relevant for your study

"The intervention target selection (i.e., which barriers to address) and message-development and refinement process was a community-led multi-phase and iterative process occurring over a two-day workshop with an interdisciplinary team: three facilitators (HST research staff members experienced in conducting HIV related trainings) and 10 participants (HST's community advisory board members, peer outreach workers, experienced in outreach and care-linkage for >5 years, and with online MSM dating apps), and HIV counseling and testing staff. The members had diverse sexual identities common in India] (gay, bisexual, kothi, panthi) [44, 45), genders (male, female, and hijra/transgender individuals), and demographic characteristics (with regards to education, age, and primary language used (English or Hindi)). Three members of the group were people living with HIV.
We used "open space technology" to facilitate communication and participation by all workshop members [46]. Open space technique is a process that has been used across disciplines to help ensure inclusion of diverse attitudes and experiences, and has been used to facilitate identification of challenges or barriers to a task or behavior (eg, HIV testing) and identification of potential solutions to overcome them. For this study, workshop members first identified challenges to HIV-testing and consistent condom use, and then mapped these to the IMB domains (i.e., the targets). The following targets were identified within the IMB Model: Information: Information about HIV transmission and prevention with condoms, logistical information (e.g., testing locations and hours); Motivation: Risk Perception, Stigma; and Behavioral Skills: how to access or make an appointment for free testing.
Next, after receiving a brief orientation to "Approach" and "Avoidance" messaging frames, participants in small groups developed short social marketing messages that could be disseminated online addressing the above-identified targets or provided solutions for overcoming the barrier (e.g., a webpage vetted to be MSM friendly listing free HIV-testing centers, which addressed lack of knowledge about safe HIV-testing venues). Workshop participants developed between 3 to 5 messages for every identified target for both Approach and Avoidance frames. Thus, we developed approximately 25–30 messages for each message Frame (Approach and Avoidance). All messages were transcreated into English or Hindi based on the original language (eg, messages initially developed in Hindi were then transcreated into English and conversely from English into Hindi). We used transcreation (as opposed to translation) to retain the essence of the original message [47], while the text was then refined using a consensus approach to further ensure comprehension and equivalency in meaning, sentiment, and framing. Next, 30 peer-staff and MSM community members at the HST drop in-center (not involved in the workshop) voted for their favorite top three messages for each factor in each of the Approach and Avoidance frames. Finally, we selected the top one or two messages receiving the most votes for each target within each frame for use in the intervention, resulting in 15 messages for each arm; eight of the messages focused on HIV-testing and

5-iii) Revisions and updating

Revisions and updating. Clearly mention the date and/or version number of the application/intervention (and comparator, if applicable) evaluated, or describe whether the intervention underwent major changes during the evaluation process, or whether the development and/or content was “frozen” during the trial. Describe dynamic components such as news feeds or changing content which may have an impact on the replicability of the intervention (for unexpected events see item 3b).

	1	2	3	4	5	
subitem not at all important	○	○	○	○	○	essential

Does your paper address subitem 5-iii?

Copy and paste relevant sections from the manuscript (include quotes in quotation marks "like this" to indicate direct quotes from your manuscript), or elaborate on this item by providing additional information not in the ms, or briefly explain why the item is not applicable/relevant for your study

Your answer

5-iv) Quality assurance methods

Provide information on quality assurance methods to ensure accuracy and quality of information provided [1], if applicable.

	1	2	3	4	5	
subitem not at all important	○	○	○	○	○	essential

Does your paper address subitem 5-iv?

Copy and paste relevant sections from the manuscript (include quotes in quotation marks "like this" to indicate direct quotes from your manuscript), or elaborate on this item by providing additional information not in the ms, or briefly explain why the item is not applicable/relevant for your study

Your answer

5-v) Ensure replicability by publishing the source code, and/or providing screenshots/screen-capture video, and/or providing flowcharts of the algorithms used

Ensure replicability by publishing the source code, and/or providing screenshots/screen-capture video, and/or providing flowcharts of the algorithms used. Replicability (i.e., other researchers should in principle be able to replicate the study) is a hallmark of scientific reporting.

	1	2	3	4	5	
subitem not at all important	○	○	●	○	○	essential

Does your paper address subitem 5-v?

Copy and paste relevant sections from the manuscript (include quotes in quotation marks "like this" to indicate direct quotes from your manuscript), or elaborate on this item by providing additional information not in the ms, or briefly explain why the item is not applicable/relevant for your study

"Examples of Messaging

Avoidance: It doesn't matter if you sleep with only 4 or 5. It only takes one. Not using condoms puts you at risk for HIV. Avoid HIV by using condoms!

Approach: Whether you ride from the front seat or back seat, you both need a helmet. Use a condom either way. Keep yourself and your partner healthy! "

5-vi) Digital preservation

Digital preservation: Provide the URL of the application, but as the intervention is likely to change or disappear over the course of the years; also make sure the intervention is archived (Internet Archive, webcitation.org, and/or publishing the source code or screenshots/videos alongside the article). As pages behind login screens cannot be archived, consider creating demo pages which are accessible without login.

	1	2	3	4	5	
subitem not at all important	◉	○	○	○	○	essential

Does your paper address subitem 5-vi?

Copy and paste relevant sections from the manuscript (include quotes in quotation marks "like this" to indicate direct quotes from your manuscript), or elaborate on this item by providing additional information not in the ms, or briefly explain why the item is not applicable/relevant for your study

Your answer

5-vii) Access

Access: Describe how participants accessed the application, in what setting/context, if they had to pay (or were paid) or not, whether they had to be a member of specific group. If known, describe how participants obtained “access to the platform and Internet” [1]. To ensure access for editors/reviewers/readers, consider to provide a “backdoor” login account or demo mode for reviewers/readers to explore the application (also important for archiving purposes, see vi).

	1	2	3	4	5	
subitem not at all important	○	○	○	○	◉	essential

Does your paper address subitem 5-vii? *

Copy and paste relevant sections from the manuscript (include quotes in quotation marks "like this" to indicate direct quotes from your manuscript), or elaborate on this item by providing additional information not in the ms, or briefly explain why the item is not applicable/relevant for your study

"From March to June, 2015, the four peer-outreach workers (two per arm) sent a standardized introductory message via the participant's chosen communication modality (ie, email, WhatsApp, or private Facebook group), followed by intervention messages two or three times per week for 12 weeks. Each set of 15 messages was sent out twice over the 12 weeks to help ensure that participants viewed them and reinforce the information contained within the messages. Thus, after the first set of 15 messages were sent out, another round of the same 15 messages was sent again. Messages for all participants were sent by the peer-outreach worker on the same days and times each week. Participants then received a final intervention message and a personal link to the follow-up survey. All messages were sent with arm-specific links to the study webpage with additional information about HIV-testing, condom use, and HST services. We compensated participants with Amazon India vouchers worth INR 300 (approximately USD $4.75) on successful completion of baseline survey and INR 400 (approximately USD $6.30) on successful

5-viii) Mode of delivery, features/functionalities/components of the intervention and comparator, and the theoretical framework

Describe mode of delivery, features/functionalities/components of the intervention and comparator, and the theoretical framework [6] used to design them (instructional strategy [1], behaviour change techniques, persuasive features, etc., see e.g., [7, 8] for terminology). This includes an in-depth description of the content (including where it is coming from and who developed it) [1]," whether [and how] it is tailored to individual circumstances and allows users to track their progress and receive feedback" [6]. This also includes a description of communication delivery channels and – if computer-mediated communication is a component – whether communication was synchronous or asynchronous [6]. It also includes information on presentation strategies [1], including page design principles, average amount of text on pages, presence of hyperlinks to other resources, etc. [1].

	1	2	3	4	5	
subitem not at all important	○	○	○	○	○	essential

Does your paper address subitem 5-viii? *

Copy and paste relevant sections from the manuscript (include quotes in quotation marks "like this" to indicate direct quotes from your manuscript), or elaborate on this item by providing additional information not in the ms, or briefly explain why the item is not applicable/relevant for your study

"The intervention target selection (i.e., which barriers to address) and message-development and refinement process was a community-led multi-phase and iterative process occurring over a two-day workshop with an interdisciplinary team: three facilitators (HST research staff members experienced in conducting HIV related trainings) and 10 participants (HST's community advisory board members, peer outreach workers, experienced in outreach and care-linkage for >5 years, and with online MSM dating apps), and HIV counseling and testing staff. The members had diverse sexual identities common in India] (gay, bisexual, kothi, panthi) [44, 45), genders (male, female, and hijra/transgender individuals), and demographic characteristics (with regards to education, age, and primary language used (English or Hindi)). Three members of the group were people living with HIV.
We used "open space technology" to facilitate communication and participation by all workshop members [46]. Open space technique is a process that has been used across disciplines to help ensure inclusion of diverse attitudes and experiences, and has been used to facilitate identification of challenges or barriers to a task or behavior (eg, HIV testing) and identification of potential solutions to overcome them. For this study, workshop members first identified challenges to HIV-testing and consistent condom use, and then mapped these to the IMB domains (i.e., the targets). The following targets were identified within the IMB Model: Information: Information about HIV transmission and prevention with condoms, logistical information (e.g., testing locations and hours); Motivation: Risk Perception, Stigma; and Behavioral Skills: how to access or make an appointment for free testing.
Next, after receiving a brief orientation to "Approach" and "Avoidance" messaging frames, participants in small groups developed short social marketing messages that could be disseminated online addressing the above-identified targets or provided solutions for overcoming the barrier (e.g., a webpage vetted to be MSM friendly listing free HIV-testing centers, which addressed lack of knowledge about safe HIV-testing venues). Workshop participants developed between 3 to 5 messages for every identified target for both Approach and Avoidance frames. Thus, we developed approximately 25–30 messages for each message Frame (Approach and Avoidance). All messages were transcreated into English or Hindi based on the original language (eg, messages initially developed in Hindi were then transcreated into English and conversely from English into Hindi). We used transcreation (as opposed to translation) to retain the essence of the original message [47], while the text was then refined using a consensus approach to further ensure comprehension and equivalency in meaning, sentiment, and framing. Next, 30 peer-staff and MSM community members at the HST drop in-center (not involved in the workshop) voted for their favorite top three messages for each factor in each of the Approach and Avoidance frames. Finally, we selected the top one or two messages receiving the most votes for each target within each frame for use in the intervention, resulting in 15 messages for each arm; eight of the messages focused on HIV-testing and

5-ix) Describe use parameters

Describe use parameters (e.g., intended "doses" and optimal timing for use). Clarify what instructions or recommendations were given to the user, e.g., regarding timing, frequency, heaviness of use, if any, or was the intervention used ad libitum.

	1	2	3	4	5	
subitem not at all important	○	○	●	○	○	essential

Does your paper address subitem 5-ix?

Copy and paste relevant sections from the manuscript (include quotes in quotation marks "like this" to indicate direct quotes from your manuscript), or elaborate on this item by providing additional information not in the ms, or briefly explain why the item is not applicable/relevant for your study

"The four peer-outreach workers (two per arm) sent a standardized introductory message via the participant's chosen communication modality (ie, email, WhatsApp, or private Facebook group), followed by intervention messages two or three times per week for 12 weeks. Each set of 15 messages was sent out twice over the 12 weeks to help ensure that participants viewed them and reinforce the information contained within the messages. Thus, after the first set of 15 messages were sent out, another round of the same 15 messages was sent again. Messages for all participants were sent by the peer-outreach worker on the same days and times each week. "

5-x) Clarify the level of human involvement

Clarify the level of human involvement (care providers or health professionals, also technical assistance) in the e-intervention or as co-intervention (detail number and expertise of professionals involved, if any, as well as "type of assistance offered, the timing and frequency of the support, how it is initiated, and the medium by which the assistance is delivered". It may be necessary to distinguish between the level of human involvement required for the trial, and the level of human involvement required for a routine application outside of a RCT setting (discuss under item 21 – generalizability).

	1	2	3	4	5	
subitem not at all important	○	○	○	●	○	essential

Does your paper address subitem 5-x?

Copy and paste relevant sections from the manuscript (include quotes in quotation marks "like this" to indicate direct quotes from your manuscript), or elaborate on this item by providing additional information not in the ms, or briefly explain why the item is not applicable/relevant for your study

As quoted from the paper in earlier subitems, peer leaders deliver messages to participant's via their chosen communication mode. Participants could communicate with their peer leader if they choose to either via email, Facebook

5-xi) Report any prompts/reminders used

Report any prompts/reminders used: Clarify if there were prompts (letters, emails, phone calls, SMS) to use the application, what triggered them, frequency etc. It may be necessary to distinguish between the level of prompts/reminders required for the trial, and the level of prompts/reminders for a routine application outside of a RCT setting (discuss under item 21 – generalizability).

	1	2	3	4	5	
subitem not at all important	○	○	○	●	○	essential

Does your paper address subitem 5-xi? *

Copy and paste relevant sections from the manuscript (include quotes in quotation marks "like this" to indicate direct quotes from your manuscript), or elaborate on this item by providing additional information not in the ms, or briefly explain why the item is not applicable/relevant for your study

"the four peer-outreach workers (two per arm) sent a standardized introductory message via the participant's chosen communication modality (ie, email, WhatsApp, or private Facebook group), followed by intervention messages two or three times per week for 12 weeks. "

5-xii) Describe any co-interventions (incl. training/support)

Describe any co-interventions (incl. training/support): Clearly state any interventions that are provided in addition to the targeted eHealth intervention, as ehealth intervention may not be designed as stand-alone intervention. This includes training sessions and support [1]. It may be necessary to distinguish between the level of training required for the trial, and the level of training for a routine application outside of a RCT setting (discuss under item 21 – generalizability.

	1	2	3	4	5	
subitem not at all important	○	○	◉	○	○	essential

Does your paper address subitem 5-xii? *

Copy and paste relevant sections from the manuscript (include quotes in quotation marks "like this" to indicate direct quotes from your manuscript), or elaborate on this item by providing additional information not in the ms, or briefly explain why the item is not applicable/relevant for your study

"HST research staff selected four MSM peer-outreach workers for the intervention who were fluent in Hindi and English and reported comfort and experience with using online dating apps, Facebook, and email, and not involved with development of the messages. Chosen peers had previously received training in HIV-related communication, community engagement, and were experienced in HIV-related outreach in Mumbai. For this pilot, the peer-outreach worker received additional specific training on online research ethics, maintaining confidentiality and privacy, and communicating via online tools.Two peer-outreach workers were randomly assigned to each arm. Peers were then randomly assigned to serve as the online peer outreach worker for half the participants within their assigned arm. Each peer was responsible for sending intervention messages to their assigned participants and to communicate with participants, if and when a participant chose to initiate any communication. There was no cross-arm communication from the peer-

6a) Completely defined pre-specified primary and secondary outcome measures, including how and when they were assessed

Does your paper address CONSORT subitem 6a? *

Copy and paste relevant sections from the manuscript (include quotes in quotation marks "like this" to indicate direct quotes from your manuscript), or elaborate on this item by providing additional information not in the ms, or briefly explain why the item is not applicable/relevant for your study

"HIV-testing. To assess the HIV-testing outcomes, we asked at baseline and follow-up “When was your last HIV test?” with response categories of “Less than 1 month ago”, “2 to 6 months ago”, “7 to 12 months ago”, “more than 12 months ago”, and “Never.” We then dichotomized responses for analysis to 6 months vs. all others, based on recommended tesging guidelines for MSM [48]. To ascertain testing intentions during and immediately after the intervention ended, we asked “Do you intend to test in the next three months?” (at baseline) and “Do you intend to test in the next month?” (at follow-up) with the answer options of Yes or No.

Consistent Condom Use. Condom use outcomes was assessed at both baseline and follow-up using the question “In the last three months, how often have you used condoms during anal sex?”, with the response options of “Always”, “Most of the time”, “Sometimes”, “Rarely” and “Never;” for analyses, we dichotomized responses as Always vs. Inconsistent (including all the other response options). "

To determine feasibility, we assessed 3 process measures: (1) enrollment data (number of individuals completing the screening survey, proportion eligible, and the proportion enrolling into the study); (2) retention (measured by a composite indicator consisting of WhatsApp and Facebook message viewed indicators, participant responses to reminder emails about completing the follow-up assessment, or completion of the follow-up assessment), and (3) completion rate (proportion of participants completing the follow-up assessment). We also assessed the relationship between completion of the follow-up assessment and baseline participant characteristics using chi-square, Fisher’s exact or t-tests as appropriate.

To determine acceptability, we tabulated the Likert-scale responses to questions about how much the group liked participating in CHALO! and used chi-square to examine differences between conditions. We thematically analyzed and coded the field notes and the brief open-ended responses on the follow-up survey based on three general categories: what was most liked, disliked, and suggestions for improving the intervention. Two team members independently coded the responses and discrepencies were resolved through discussion.

To determine early efficacy, we undertook several steps. We first described the sample using frequencies and means, and examined potential differences in baseline characteristics and behaviors using chi-square and t-tests, as appropriate. To assess the intervention’s early impact, we examined potential changes in four outcomes ((1) composite of HIV-testing plus Intention to test (2) HIV-testing alone, (3) intention to test for HIV, and (4) consistent condom use), we first compared the two arms among those completing the post-intervention assessment using chi-square tests. Next, we conducted a pooled pre-post analysis (i.e., within-subjects, across time) for HIV-testing, intention to test for HIV, and consistent condom use using Mcnemer’s test.

6a-i) Online questionnaires: describe if they were validated for online use and apply CHERRIES items to describe how the questionnaires were designed/deployed

If outcomes were obtained through online questionnaires, describe if they were validated for online use and apply CHERRIES items to describe how the questionnaires were designed/deployed [9].

	1	2	3	4	5	
subitem not at all important	◉	○	○	○	○	essential

Does your paper address subitem 6a-i?

Copy and paste relevant sections from manuscript text

The online questionnaires were pilot and refine with the target audience.

6a-ii) Describe whether and how "use" (including intensity of use/dosage) was defined/measured/monitored

Describe whether and how "use" (including intensity of use/dosage) was defined/measured/monitored (logins, logfile analysis, etc.). Use/adoption metrics are important process outcomes that should be reported in any ehealth trial.

	1	2	3	4	5	
subitem not at all important	○	○	○	○	○	essential

Does your paper address subitem 6a-ii?

Copy and paste relevant sections from manuscript text

Your answer

6a-iii) Describe whether, how, and when qualitative feedback from participants was obtained

Describe whether, how, and when qualitative feedback from participants was obtained (e.g., through emails, feedback forms, interviews, focus groups).

	1	2	3	4	5	
subitem not at all important	○	○	○	◉	○	essential

Does your paper address subitem 6a-iii?

Copy and paste relevant sections from manuscript text

"Qualitative Feedback. To evaluate acceptability, identify implementation challenges, and elicit suggestions to refine CHALO!, we collected field notes from 1) our weekly project meetings with the research team, 2) peer-outreach staff during and at the end of study, and 3) two focus groups of CHALO! participants (N=6 to 8 per group) after intervention completion. Peer intervention staff also elicited feedback via email, WhatsApp, or Facebook Messenger from those not completing the follow-up assessment to evaluate reasons for survey non-completion. "

6b) Any changes to trial outcomes after the trial commenced, with reasons

Does your paper address CONSORT subitem 6b? *

Copy and paste relevant sections from the manuscript (include quotes in quotation marks "like this" to indicate direct quotes from your manuscript), or elaborate on this item by providing additional information not in the ms, or briefly explain why the item is not applicable/relevant for your study

There were no changes to trial outcomes after the trial commenced

7a) How sample size was determined

NPT: When applicable, details of whether and how the clustering by care provides or centers was addressed

7a-i) Describe whether and how expected attrition was taken into account when calculating the sample size

Describe whether and how expected attrition was taken into account when calculating the sample size.

	1	2	3	4	5	
subitem not at all important	○	○	○	○	○	essential

Does your paper address subitem 7a-i?

Copy and paste relevant sections from manuscript title (include quotes in quotation marks "like this" to indicate direct quotes from your manuscript), or elaborate on this item by providing additional information not in the ms, or briefly explain why the item is not applicable/relevant for your study

Your answer

7b) When applicable, explanation of any interim analyses and stopping guidelines

Does your paper address CONSORT subitem 7b? *

Copy and paste relevant sections from the manuscript (include quotes in quotation marks "like this" to indicate direct quotes from your manuscript), or elaborate on this item by providing additional information not in the ms, or briefly explain why the item is not applicable/relevant for your study

There are no applicable interim analyses or stopping guidelines

8a) Method used to generate the random allocation sequence

NPT: When applicable, how care providers were allocated to each trial group

Does your paper address CONSORT subitem 8a? *

Copy and paste relevant sections from the manuscript (include quotes in quotation marks "like this" to indicate direct quotes from your manuscript), or elaborate on this item by providing additional information not in the ms, or briefly explain why the item is not applicable/relevant for your study

Please see 8b for randomization details.

8b) Type of randomisation; details of any restriction (such as blocking and block size)

Does your paper address CONSORT subitem 8b? *

Copy and paste relevant sections from the manuscript (include quotes in quotation marks "like this" to indicate direct quotes from your manuscript), or elaborate on this item by providing additional information not in the ms, or briefly explain why the item is not applicable/relevant for your study

Peer Leaders ".Two peer-outreach workers were randomly assigned to each arm. Peers were then randomly assigned to serve as the online peer outreach worker for half the participants within their assigned arm. Each peer was responsible for sending intervention messages to their assigned participants and to communicate with participants, if and when a participant chose to initiate any communication. There was no cross-arm communication from the peer-outreach workers to participants in the arm to which they were not assigned."

Participants: " . After confirming contact information provided, participants were randomized 1:1 to either the Approach or Avoidance framed conditions."

9) Mechanism used to implement the random allocation sequence (such as sequentially numbered containers), describing any steps taken to conceal the sequence until interventions were assigned

Does your paper address CONSORT subitem 9? *

Copy and paste relevant sections from the manuscript (include quotes in quotation marks "like this" to indicate direct quotes from your manuscript), or elaborate on this item by providing additional information not in the ms, or briefly explain why the item is not applicable/relevant for your study

Not applicable.

10) Who generated the random allocation sequence, who enrolled participants, and who assigned participants to interventions

Does your paper address CONSORT subitem 10? *

Copy and paste relevant sections from the manuscript (include quotes in quotation marks "like this" to indicate direct quotes from your manuscript), or elaborate on this item by providing additional information not in the ms, or briefly explain why the item is not applicable/relevant for your study

Random allocation sequence was computer generated by the lead author. Participants were enrolled by a Research Assistant and the RA also assigned participants to the intervention.

11a) If done, who was blinded after assignment to interventions (for example, participants, care providers, those assessing outcomes) and how

NPT: Whether or not administering co-interventions were blinded to group assignment

11a-i) Specify who was blinded, and who wasn’t

Specify who was blinded, and who wasn’t. Usually, in web-based trials it is not possible to blind the participants [1, 3] (this should be clearly acknowledged), but it may be possible to blind outcome assessors, those doing data analysis or those administering co-interventions (if any).

	1	2	3	4	5	
subitem not at all important	○	○	○	◉	○	essential

Does your paper address subitem 11a-i? *

Copy and paste relevant sections from the manuscript (include quotes in quotation marks "like this" to indicate direct quotes from your manuscript), or elaborate on this item by providing additional information not in the ms, or briefly explain why the item is not applicable/relevant for your study

The participants were blinded since they were randomly assigned to either of the conditions, with no knowledge of another arm. The statistician was also blinded.

11a-ii) Discuss e.g., whether participants knew which intervention was the “intervention of interest” and which one was the “comparator”

Informed consent procedures (4a-ii) can create biases and certain expectations - discuss e.g., whether participants knew which intervention was the “intervention of interest” and which one was the “comparator”.

	1	2	3	4	5	
subitem not at all important	○	○	○	◉	○	essential

Does your paper address subitem 11a-ii?

Copy and paste relevant sections from the manuscript (include quotes in quotation marks "like this" to indicate direct quotes from your manuscript), or elaborate on this item by providing additional information not in the ms, or briefly explain why the item is not applicable/relevant for your study

Participants were not aware of another arm

11b) If relevant, description of the similarity of interventions

(this item is usually not relevant for ehealth trials as it refers to similarity of a placebo or sham intervention to a active medication/intervention)

Does your paper address CONSORT subitem 11b? *

Copy and paste relevant sections from the manuscript (include quotes in quotation marks "like this" to indicate direct quotes from your manuscript), or elaborate on this item by providing additional information not in the ms, or briefly explain why the item is not applicable/relevant for your study

This is not relevant.

12a) Statistical methods used to compare groups for primary and secondary outcomes

NPT: When applicable, details of whether and how the clustering by care providers or centers was addressed

Does your paper address CONSORT subitem 12a? *

Copy and paste relevant sections from the manuscript (include quotes in quotation marks "like this" to indicate direct quotes from your manuscript), or elaborate on this item by providing additional information not in the ms, or briefly explain why the item is not applicable/relevant for your study

"To determine feasibility, we assessed 3 process measures: (1) enrollment data (number of individuals completing the screening survey, proportion eligible, and the proportion enrolling into the study); (2) retention (measured by a composite indicator consisting of WhatsApp and Facebook message viewed indicators, participant responses to reminder emails about completing the follow-up assessment, or completion of the follow-up assessment), and (3) completion rate (proportion of participants completing the follow-up assessment). We also assessed the relationship between completion of the follow-up assessment and baseline participant characteristics using chi-square, Fisher's exact or t-tests as appropriate.

To determine acceptability, we tabulated the Likert-scale responses to questions about how much the group liked participating in CHALO! and used chi-square to examine differences between conditions. We thematically analyzed and coded the field notes and the brief open-ended responses on the follow-up survey based on three general categories: what was most liked, disliked, and suggestions for improving the intervention. Two team members independently coded the responses and discrepencies were resolved through discussion.

To determine early efficacy, we undertook several steps. We first described the sample using frequencies and means, and examined potential differences in baseline characteristics and behaviors using chi-square and t-tests, as appropriate. To assess the intervention's early impact, we examined potential changes in four outcomes ((1) composite of HIV-testing plus Intention to test (2) HIV-testing alone, (3) intention to test for HIV, and (4) consistent condom use), we first compared the two arms among those completing the post-intervention assessment using chi-square tests. Next, we conducted a pooled pre-post analysis (i.e., within-subjects, across time) for HIV-testing, intention to test for HIV, and consistent condom use using Mcnemer's test. "

12a-i) Imputation techniques to deal with attrition / missing values

Imputation techniques to deal with attrition / missing values: Not all participants will use the intervention/comparator as intended and attrition is typically high in ehealth trials. Specify how participants who did not use the application or dropped out from the trial were treated in the statistical analysis (a complete case analysis is strongly discouraged, and simple imputation techniques such as LOCF may also be problematic [4]).

	1	2	3	4	5	
subitem not at all important	○	○	○	◉	○	essential

Does your paper address subitem 12a-i? *

Copy and paste relevant sections from the manuscript (include quotes in quotation marks "like this" to indicate direct quotes from your manuscript), or elaborate on this item by providing additional information not in the ms, or briefly explain why the item is not applicable/relevant for your study

" There were no significant differences between conditions in the proportion of participants retained, accessing the follow-up assessment, and completing the follow-up assessment. There were also no significant differences in baseline demographic and behavioral characteristics among those retained or completing the follow-up assessment, except in regards to sexual orientation (Supplementry Table)."

12b) Methods for additional analyses, such as subgroup analyses and adjusted analyses

Does your paper address CONSORT subitem 12b? *

Copy and paste relevant sections from the manuscript (include quotes in quotation marks "like this" to indicate direct quotes from your manuscript), or elaborate on this item by providing additional information not in the ms, or briefly explain why the item is not applicable/relevant for your study

This is not applicable. Analysis have been previously described in earlier subitems.

X26) REB/IRB Approval and Ethical Considerations [recommended as subheading under "Methods"] (not a CONSORT item)

X26-i) Comment on ethics committee approval

	1	2	3	4	5	
subitem not at all important	○	○	○	○	○	essential

Does your paper address subitem X26-i?

Copy and paste relevant sections from the manuscript (include quotes in quotation marks "like this" to indicate direct quotes from your manuscript), or elaborate on this item by providing additional information not in the ms, or briefly explain why the item is not applicable/relevant for your study

Your answer

x26-ii) Outline informed consent procedures

Outline informed consent procedures e.g., if consent was obtained offline or online (how? Checkbox, etc.?), and what information was provided (see 4a-ii). See [6] for some items to be included in informed consent documents.

	1	2	3	4	5	
subitem not at all important	○	○	○	●	○	essential

Does your paper address subitem X26-ii?

Copy and paste relevant sections from the manuscript (include quotes in quotation marks "like this" to indicate direct quotes from your manuscript), or elaborate on this item by providing additional information not in the ms, or briefly explain why the item is not applicable/relevant for your study

"Potential participants clicked through the ads to complete an online consent and eligibility screener; if eligible, they automatically continued on to the baseline survey. "

X26-iii) Safety and security procedures

Safety and security procedures, incl. privacy considerations, and any steps taken to reduce the likelihood or detection of harm (e.g., education and training, availability of a hotline)

	1	2	3	4	5	
subitem not at all important	○	○	○	●	○	essential

Does your paper address subitem X26-iii?

Copy and paste relevant sections from the manuscript (include quotes in quotation marks "like this" to indicate direct quotes from your manuscript), or elaborate on this item by providing additional information not in the ms, or briefly explain why the item is not applicable/relevant for your study

"For this pilot, the peer-outreach worker received additional specific training on online research ethics, maintaining confidentiality and privacy, and communicating via online tools"

RESULTS

13a) For each group, the numbers of participants who were randomly assigned, received intended treatment, and were analysed for the primary outcome

NPT: The number of care providers or centers performing the intervention in each group and the number of patients treated by each care provider in each center

Does your paper address CONSORT subitem 13a? *

Copy and paste relevant sections from the manuscript (include quotes in quotation marks "like this" to indicate direct quotes from your manuscript), or elaborate on this item by providing additional information not in the ms, or briefly explain why the item is not applicable/relevant for your study

Figure 1 and Table 1 present this information. A total of 244 participant were randomized and analyses in the analysis.

13b) For each group, losses and exclusions after randomisation, together with reasons

Does your paper address CONSORT subitem 13b? (NOTE: Preferably, this is shown in a CONSORT flow diagram) *

Copy and paste relevant sections from the manuscript (include quotes in quotation marks "like this" to indicate direct quotes from your manuscript), or elaborate on this item by providing additional information not in the ms, or briefly explain why the item is not applicable/relevant for your study

"Overall, 200 of the 244 enrolled participants (82%) were retained through the end of the intervention (Figure 1). The post-intervention assessment link was clicked on by 153 (63%) participants and was completed by 130 (53%) participants. There were no significant differences between conditions in the proportion of participants retained, accessing the follow-up assessment, and completing the follow-up assessment. There were also no significant differences in baseline demographic and behavioral characteristics among those retained or completing the follow-up assessment, except in regards to sexual orientation (Supplementry Table). "

13b-i) Attrition diagram

Strongly recommended: An attrition diagram (e.g., proportion of participants still logging in or using the intervention/comparator in each group plotted over time, similar to a survival curve) or other figures or tables demonstrating usage/dose/engagement.

	1	2	3	4	5	
subitem not at all important	○	○	○	◉	○	essential

Does your paper address subitem 13b-i?

Copy and paste relevant sections from the manuscript or cite the figure number if applicable (include quotes in quotation marks "like this" to indicate direct quotes from your manuscript), or elaborate on this item by providing additional information not in the ms, or briefly explain why the item is not applicable/relevant for your study

Please refer to figure 1 in the manuscript

14a) Dates defining the periods of recruitment and follow-up

Does your paper address CONSORT subitem 14a? *

Copy and paste relevant sections from the manuscript (include quotes in quotation marks "like this" to indicate direct quotes from your manuscript), or elaborate on this item by providing additional information not in the ms, or briefly explain why the item is not applicable/relevant for your study

recruitment: "from February to March 2015, recruitment advertisements were disseminated"

Intervention/follow-up: ",from March to June, 2015, the four peer-outreach workers (two per arm) sent a standardized introductory message via the participant’s chosen communication modality"

14a-i) Indicate if critical “secular events” fell into the study period

Indicate if critical “secular events” fell into the study period, e.g., significant changes in Internet resources available or “changes in computer hardware or Internet delivery resources”

	1	2	3	4	5	
subitem not at all important	(•)	( )	( )	( )	( )	essential

Does your paper address subitem 14a-i?

Copy and paste relevant sections from the manuscript (include quotes in quotation marks "like this" to indicate direct quotes from your manuscript), or elaborate on this item by providing additional information not in the ms, or briefly explain why the item is not applicable/relevant for your study

This is not applicable

14b) Why the trial ended or was stopped (early)

Does your paper address CONSORT subitem 14b? *

Copy and paste relevant sections from the manuscript (include quotes in quotation marks "like this" to indicate direct quotes from your manuscript), or elaborate on this item by providing additional information not in the ms, or briefly explain why the item is not applicable/relevant for your study

The trial did not end or stop early

15) A table showing baseline demographic and clinical characteristics for each group

NPT: When applicable, a description of care providers (case volume, qualification, expertise, etc.) and centers (volume) in each group

Does your paper address CONSORT subitem 15? *

Copy and paste relevant sections from the manuscript (include quotes in quotation marks "like this" to indicate direct quotes from your manuscript), or elaborate on this item by providing additional information not in the ms, or briefly explain why the item is not applicable/relevant for your study

Table 1 presents baseline demographic characteristics for each group.

15-i) Report demographics associated with digital divide issues

In ehealth trials it is particularly important to report demographics associated with digital divide issues, such as age, education, gender, social-economic status, computer/Internet/ehealth literacy of the participants, if known.

	1	2	3	4	5	
subitem not at all important	○	○	○	●	○	essential

Does your paper address subitem 15-i? *

Copy and paste relevant sections from the manuscript (include quotes in quotation marks "like this" to indicate direct quotes from your manuscript), or elaborate on this item by providing additional information not in the ms, or briefly explain why the item is not applicable/relevant for your study

Demographics associated with digital divide issues are presented in table.

16) For each group, number of participants (denominator) included in each analysis and whether the analysis was by original assigned groups

16-i) Report multiple "denominators" and provide definitions

Report multiple "denominators" and provide definitions: Report N's (and effect sizes) "across a range of study participation [and use] thresholds" [1], e.g., N exposed, N consented, N used more than x times, N used more than y weeks, N participants "used" the intervention/comparator at specific pre-defined time points of interest (in absolute and relative numbers per group). Always clearly define "use" of the intervention.

	1	2	3	4	5	
subitem not at all important	○	○	○	◉	○	essential

Does your paper address subitem 16-i? *

Copy and paste relevant sections from the manuscript (include quotes in quotation marks "like this" to indicate direct quotes from your manuscript), or elaborate on this item by providing additional information not in the ms, or briefly explain why the item is not applicable/relevant for your study

All denominators are included (please refer to table 1 and table 2).

16-ii) Primary analysis should be intent-to-treat

Primary analysis should be intent-to-treat, secondary analyses could include comparing only "users", with the appropriate caveats that this is no longer a randomized sample (see 18-i).

	1	2	3	4	5	
subitem not at all important	○	○	○	○	○	essential

Does your paper address subitem 16-ii?

Copy and paste relevant sections from the manuscript (include quotes in quotation marks "like this" to indicate direct quotes from your manuscript), or elaborate on this item by providing additional information not in the ms, or briefly explain why the item is not applicable/relevant for your study

Your answer

17a) For each primary and secondary outcome, results for each group, and the estimated effect size and its precision (such as 95% confidence interval)

Does your paper address CONSORT subitem 17a? *

Copy and paste relevant sections from the manuscript (include quotes in quotation marks "like this" to indicate direct quotes from your manuscript), or elaborate on this item by providing additional information not in the ms, or briefly explain why the item is not applicable/relevant for your study

For primary and secondary outcomes N (%) and p value are presented. Please refer to table 2.

17a-i) Presentation of process outcomes such as metrics of use and intensity of use

In addition to primary/secondary (clinical) outcomes, the presentation of process outcomes such as metrics of use and intensity of use (dose, exposure) and their operational definitions is critical. This does not only refer to metrics of attrition (13-b) (often a binary variable), but also to more continuous exposure metrics such as “average session length”. These must be accompanied by a technical description how a metric like a “session” is defined (e.g., timeout after idle time) [1] (report under item 6a).

	1	2	3	4	5	
subitem not at all important	○	○	○	○	○	essential

Does your paper address subitem 17a-i?

Copy and paste relevant sections from the manuscript (include quotes in quotation marks "like this" to indicate direct quotes from your manuscript), or elaborate on this item by providing additional information not in the ms, or briefly explain why the item is not applicable/relevant for your study

This is not applicable

17b) For binary outcomes, presentation of both absolute and relative effect sizes is recommended

Does your paper address CONSORT subitem 17b? *

Copy and paste relevant sections from the manuscript (include quotes in quotation marks "like this" to indicate direct quotes from your manuscript), or elaborate on this item by providing additional information not in the ms, or briefly explain why the item is not applicable/relevant for your study

This is not applicable.

18) Results of any other analyses performed, including subgroup analyses and adjusted analyses, distinguishing pre-specified from exploratory

Does your paper address CONSORT subitem 18? *

Copy and paste relevant sections from the manuscript (include quotes in quotation marks "like this" to indicate direct quotes from your manuscript), or elaborate on this item by providing additional information not in the ms, or briefly explain why the item is not applicable/relevant for your study

Analysis of potential contamination is presented in the results section.

18-i) Subgroup analysis of comparing only users

A subgroup analysis of comparing only users is not uncommon in ehealth trials, but if done, it must be stressed that this is a self-selected sample and no longer an unbiased sample from a randomized trial (see 16-iii).

	1	2	3	4	5	
subitem not at all important	○	○	○	○	○	essential

Does your paper address subitem 18-i?

Copy and paste relevant sections from the manuscript (include quotes in quotation marks "like this" to indicate direct quotes from your manuscript), or elaborate on this item by providing additional information not in the ms, or briefly explain why the item is not applicable/relevant for your study

Your answer

19) All important harms or unintended effects in each group

(for specific guidance see CONSORT for harms)

Does your paper address CONSORT subitem 19? *

Copy and paste relevant sections from the manuscript (include quotes in quotation marks "like this" to indicate direct quotes from your manuscript), or elaborate on this item by providing additional information not in the ms, or briefly explain why the item is not applicable/relevant for your study

There were no unintended effects.

19-i) Include privacy breaches, technical problems

Include privacy breaches, technical problems. This does not only include physical “harm” to participants, but also incidents such as perceived or real privacy breaches [1], technical problems, and other unexpected/unintended incidents. “Unintended effects” also includes unintended positive effects [2].

	1	2	3	4	5	
subitem not at all important	○	○	○	○	○	essential

Does your paper address subitem 19-i?

Copy and paste relevant sections from the manuscript (include quotes in quotation marks "like this" to indicate direct quotes from your manuscript), or elaborate on this item by providing additional information not in the ms, or briefly explain why the item is not applicable/relevant for your study

Your answer

19-ii) Include qualitative feedback from participants or observations from staff/researchers

Include qualitative feedback from participants or observations from staff/researchers, if available, on strengths and shortcomings of the application, especially if they point to unintended/unexpected effects or uses. This includes (if available) reasons for why people did or did not use the application as intended by the developers.

	1	2	3	4	5	
subitem not at all important	○	○	○	◉	○	essential

Does your paper address subitem 19-ii?

Copy and paste relevant sections from the manuscript (include quotes in quotation marks "like this" to indicate direct quotes from your manuscript), or elaborate on this item by providing additional information not in the ms, or briefly explain why the item is not applicable/relevant for your study

"Content analysis of free-text responses about what participants liked most about CHALO! revealed the following themes: the intervention was useful and provided supportive information; messages were engaging or motivating; created a sense of community and acceptance; and made them feel good about helping their community by participating in the study. In regards to what participants least liked about CHALO!, individuals reported the survey was too long or redundant, felt that messages were not frequent enough, or had comments pertaining to the graphical appearance of the messages. Suggestions for improvement included having a larger social media presence, continuing the messaging for a longer duration, expanding topics, and using audio-visual or interactive graphics (e.g., video-clips). Field notes and feedback from peer-outreach staff indicated that the most common reason for not completing the follow-up assessment was not finding the Amazon.co.in incentive useful."

DISCUSSION

22) Interpretation consistent with results, balancing benefits and harms, and considering other relevant evidence

NPT: In addition, take into account the choice of the comparator, lack of or partial blinding, and unequal expertise of care providers or centers in each group

22-i) Restate study questions and summarize the answers suggested by the data, starting with primary outcomes and process outcomes (use)

Restate study questions and summarize the answers suggested by the data, starting with primary outcomes and process outcomes (use).

	1	2	3	4	5	
subitem not at all important	○	○	○	◉	○	essential

Does your paper address subitem 22-i? *

Copy and paste relevant sections from the manuscript (include quotes in quotation marks "like this" to indicate direct quotes from your manuscript), or elaborate on this item by providing additional information not in the ms, or briefly explain why the item is not applicable/relevant for your study

"Using a community based participatory research process, we developed and implemented an Internet-based HIV prevention intervention for MSM in India. Findings from this pilot study showed that the CHALO! intervention delivery model was feasible to implement by a community based organization, and acceptable to participants, particularly those identifying as gay/homosexual. The intervention also demonstrated preliminary evidence for improving HIV-testing and intention to test for HIV across both trial arms by self-report, with a greater increase in the Avoidance-framed arm. However, the intervention had no impact on condom use."

22-ii) Highlight unanswered new questions, suggest future research

Highlight unanswered new questions, suggest future research.

	1	2	3	4	5	
subitem not at all important	○	○	○	●	○	essential

Does your paper address subitem 22-ii?

Copy and paste relevant sections from the manuscript (include quotes in quotation marks "like this" to indicate direct quotes from your manuscript), or elaborate on this item by providing additional information not in the ms, or briefly explain why the item is not applicable/relevant for your study

"Fully powered Internet-based intervention studies are warranted to examine the impact of these online models with more objective measures of HIV-testing, as well as assessment of downstream outcomes of linkage to care (for both treatment and prevention). Additionally, HIV prevention studies of longer duration of fully online interventions in India and other low-income settings are needed to understand long term retention and program effectiveness. Unlike online interventions which rely on specific software platforms or require high technical expertise and resources, the CHALO! intervention model – including the rigorous community based development process for message creation – may also serve as a model for future ICT-based interventions that are able to accommodate the constantly shifting sociotechnical

20) Trial limitations, addressing sources of potential bias, imprecision, and, if relevant, multiplicity of analyses

20-i) Typical limitations in ehealth trials

Typical limitations in ehealth trials: Participants in ehealth trials are rarely blinded. Ehealth trials often look at a multiplicity of outcomes, increasing risk for a Type I error. Discuss biases due to non-use of the intervention/usability issues, biases through informed consent procedures, unexpected events.

	1	2	3	4	5	
subitem not at all important	○	○	○	◉	○	essential

Does your paper address subitem 20-i? *

Copy and paste relevant sections from the manuscript (include quotes in quotation marks "like this" to indicate direct quotes from your manuscript), or elaborate on this item by providing additional information not in the ms, or briefly explain why the item is not applicable/relevant for your study

"This study should be interpreted in light of its limitations. First, our measures were self-report, which may have introduced social desirability bias. However, given that we only observed changes in HIV-testing and not for condom use, as well as the relatively anonymous nature of participant enrollment, social desirability may have played a limited role. Future studies with more objective measures are nevertheless needed. Second, this study recruited MSM online who reported living in Mumbai, and thus may not be generalizable to online MSM elsewhere in India, particularly in settings which may not have MSM-sensitive physical services or a wide range of HIV-testing sites available. Third, men who identified as bisexual and straight had low retention, indicating this pilot intervention likely had minimal impact on these groups, who may be at higher risk for HIV.[65, 66] Future Internet based interventions for HIV prevention may benefit from taking into account sexual identity and tailoring contents specific to bisexual and straight identified MSM. Finally, there could have been potential contamination between study arms, given that a quarter of participants reported sharing the digital messages and a quarter knew of others participating in the study. "

21) Generalisability (external validity, applicability) of the trial findings

NPT: External validity of the trial findings according to the intervention, comparators, patients, and care providers or centers involved in the trial

21-i) Generalizability to other populations

Generalizability to other populations: In particular, discuss generalizability to a general Internet population, outside of a RCT setting, and general patient population, including applicability of the study results for other organizations

	1	2	3	4	5	
subitem not at all important	○	○	○	◉	○	essential

Does your paper address subitem 21-i?

Copy and paste relevant sections from the manuscript (include quotes in quotation marks "like this" to indicate direct quotes from your manuscript), or elaborate on this item by providing additional information not in the ms, or briefly explain why the item is not applicable/relevant for your study

"Our pilot study extends the literature by demonstrating the potential utility of a peer-delivered messaging intervention in a low-income country setting. Our findings also support the feasibility of implimenting online interventions for MSM in India, with other recent data showing the ability to rapidly engage diverse Indian MSM online, including in rural areas"

21-ii) Discuss if there were elements in the RCT that would be different in a routine application setting

Discuss if there were elements in the RCT that would be different in a routine application setting (e.g., prompts/reminders, more human involvement, training sessions or other co-interventions) and what impact the omission of these elements could have on use, adoption, or outcomes if the intervention is applied outside of a RCT setting.

	1	2	3	4	5	
subitem not at all important	○	○	○	○	○	essential

Does your paper address subitem 21-ii?

Copy and paste relevant sections from the manuscript (include quotes in quotation marks "like this" to indicate direct quotes from your manuscript), or elaborate on this item by providing additional information not in the ms, or briefly explain why the item is not applicable/relevant for your study

Your answer

OTHER INFORMATION

23) Registration number and name of trial registry

Does your paper address CONSORT subitem 23? *

Copy and paste relevant sections from the manuscript (include quotes in quotation marks "like this" to indicate direct quotes from your manuscript), or elaborate on this item by providing additional information not in the ms, or briefly explain why the item is not applicable/relevant for your study

This is not applicable

24) Where the full trial protocol can be accessed, if available

Does your paper address CONSORT subitem 24? *

Cite a Multimedia Appendix, other reference, or copy and paste relevant sections from the manuscript (include quotes in quotation marks "like this" to indicate direct quotes from your manuscript), or elaborate on this item by providing additional information not in the ms, or briefly explain why the item is not applicable/relevant for your study

This is not applicable.

25) Sources of funding and other support (such as supply of drugs), role of funders

Does your paper address CONSORT subitem 25? *

Copy and paste relevant sections from the manuscript (include quotes in quotation marks "like this" to indicate direct quotes from your manuscript), or elaborate on this item by providing additional information not in the ms, or briefly explain why the item is not applicable/relevant for your study

"This study was supported in part by a pilot grant from the Albert Einstein College of Medicine's Global Health Center, NIH K23MH102118 (PI Viraj V. Patel), the Einstein-Rockefeller-CUNY Center for AIDS Research (NIH P30AI051519), and NIH R25DA023021 (PI Julia H. Arnsten). The funding sources had no role in study design, conduct of the study, data collection or analysis, interpretation of results, manuscript preparation, or descision to submit the manuscript for publication. "

X27) Conflicts of Interest (not a CONSORT item)

X27-i) State the relation of the study team towards the system being evaluated

In addition to the usual declaration of interests (financial or otherwise), also state the relation of the study team towards the system being evaluated, i.e., state if the authors/evaluators are distinct from or identical with the developers/sponsors of the intervention.

	1	2	3	4	5	
subitem not at all important	○	○	○	○	○	essential

Does your paper address subitem X27-i?

Copy and paste relevant sections from the manuscript (include quotes in quotation marks "like this" to indicate direct quotes from your manuscript), or elaborate on this item by providing additional information not in the ms, or briefly explain why the item is not applicable/relevant for your study

Your answer

About the CONSORT EHEALTH checklist

As a result of using this checklist, did you make changes in your manuscript? *

- ○ yes, major changes
- ○ yes, minor changes
- ◉ no

What were the most important changes you made as a result of using this checklist?

Your answer

How much time did you spend on going through the checklist INCLUDING making changes in your manuscript *

7

As a result of using this checklist, do you think your manuscript has improved? *

- ○ yes
- ◉ no
- ○ Other:

Would you like to become involved in the CONSORT EHEALTH group?

This would involve for example becoming involved in participating in a workshop and writing an "Explanation and Elaboration" document

- ○ yes
- ◉ no
- ○ Other:

Any other comments or questions on CONSORT EHEALTH

Your answer

STOP - Save this form as PDF before you click submit

To generate a record that you filled in this form, we recommend to generate a PDF of this page (on a Mac, simply select "print" and then select "print as PDF") before you submit it.

When you submit your (revised) paper to JMIR, please upload the PDF as supplementary file.

Don't worry if some text in the textboxes is cut off, as we still have the complete information in our database. Thank you!

Final step: Click submit !

Click submit so we have your answers in our database!

Submit

Never submit passwords through Google Forms.

This content is neither created nor endorsed by Google. Report Abuse - Terms of Service - Privacy Policy

Google Forms
